# A Comprehensive Review of Bio-Inspired Surface and Morphological Structures for Enhancing Aerodynamic and Hydrodynamic Performances: Trends, Mechanisms, and Future Horizons

**DOI:** 10.3390/biomimetics11080523

**Published:** 2026-07-23

**Authors:** Masuruddin Shaik, Wei-Xi Huang

**Affiliations:** AML & LASP, School of Aerospace Engineering, Tsinghua University, Haidain District, Beijing 100190, China; masruddin.sk@gmail.com

**Keywords:** biomimetic surfaces, sharkskin denticles, lotus leaf effect, fish scale textures, superhydrophobic coatings, vegetation canopy, flexible filament

## Abstract

Bio-inspired wing designs have emerged as a revolutionary approach to enhancing aerodynamic and hydrodynamic performance across a wide range of engineering applications. This review presents a comprehensive exploration of the biological principles, physical mechanisms, and performance outcomes associated with aerodynamic characteristics of aerial vehicles through fine-scale biomimetic features. This review categorizes these bio-inspired surface and morphological models into static, superhydrophobic, and dynamic smart structures. Furthermore, it examines the underlying fluid mechanics through both experimental and computational studies, detailing key mechanisms including vortex manipulation, transition delay, reconfiguration, wake stabilization and flow deceleration. While demonstrating strong potential, the field faces challenges in scalability, structural integration, environmental durability, and performance under variable flow conditions. Future directions emphasize multifunctionality and standardized validation protocols. By unifying insights from nature with advances in biomimetics and fluid dynamics, this review outlines a roadmap for the next generation of intelligent, high-performance aerodynamic and hydrodynamic systems.

## 1. Introduction

Aerodynamic and hydrodynamic performance enhancements have long been a central challenge in both aerospace and marine engineering due to their direct influences on energy consumption, performance efficiency, and structural wear. As fluid flow interacts with solid bodies, the surface friction and pressure gradients result in parasitic drag, which limits velocity, range, and fuel efficiency of vehicles [1]. In the aerospace industry, even a minor reduction in drag can lead to significant fuel savings, directly affecting operational costs and environmental emissions [2,3]. Similarly, in marine applications, drag reduction translates into improved propulsion efficiency and reduced cavitation damage [4]. Traditionally, this issue has been tackled using smooth surfaces, streamlined shapes, and active control mechanisms like suction, blowing, or plasma actuators [5,6]. However, these methods often involve complex systems and energy penalties, motivating the exploration of more efficient passive strategies.

**Figure 1:**
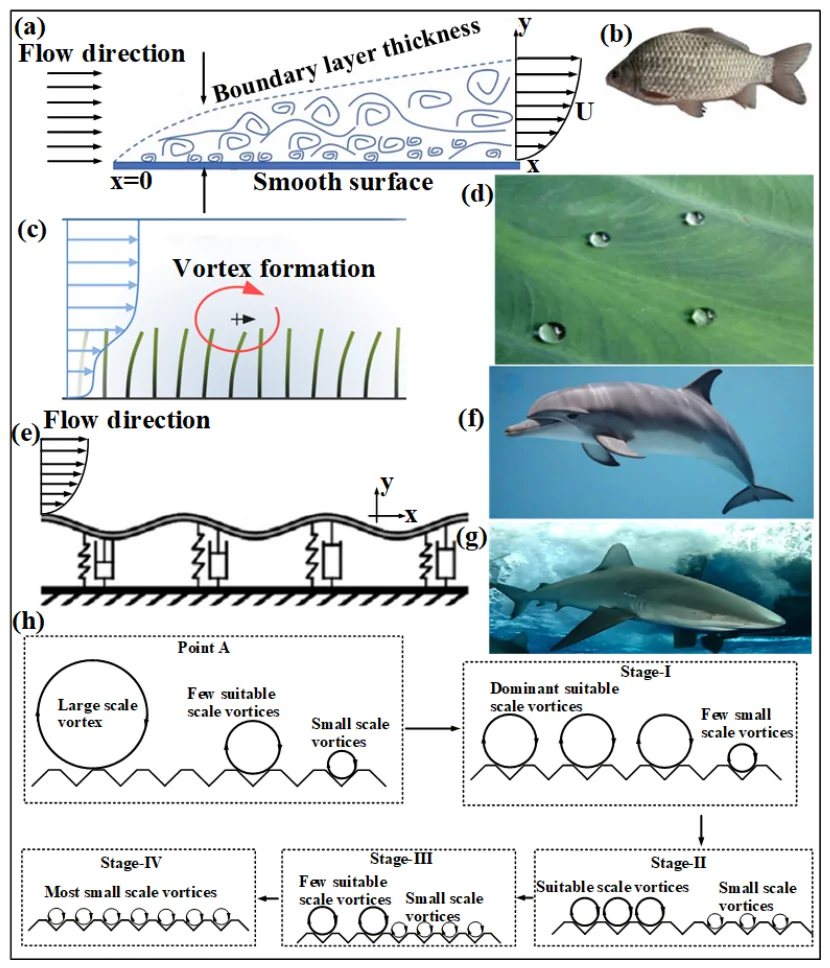
Bio-inspired drag reduction methods: (**a**) Fully developed turbulent flow [12]. (**b**) Optical illustration of fish swimming [9]. (**c**) Dominant turbulence on canopy [13]. (**d**) Illustration of water slipping on lotus leaf [12]. (**e**) Numerical modeling of compliant wall [14]. (**f**) Optical illustration of dolphin swimming [12]. (**g**) Shark swimming in ocean [15]. (**h**) The evolution and diffusion of large scale vortices into small scale vortices [9] (U, streamwise flow; **x**, flow direction; **y**, normal direction).

Biomimicry, the practice of drawing design inspiration from biological systems, has led to revolutionary breakthroughs in surface engineering [7,8]. Nature, through millennia of evolutionary pressure, has developed a wide array of organisms that navigate fluid environments with exceptional efficiency. From the ribbed skin of sharks that minimizes skin friction to the self-cleaning micro-/nano-texture of lotus leaves, these natural solutions offer unique insights into passive and adaptive drag management. A schematic representation of these biological inspirations are depicted in Figure 1. Studying these biological phenomena enables engineers to abstract functional principles that can be implemented in engineered systems. Importantly, biomimicry involves decoding the underlying biomechanical logic and reinterpreting it through the lens of modern technology and material science. The significance of fine structures in micro- and nano-scale surface features is increasingly evident in the way they interact with fluid flow. These structures, although small in size, play a disproportionate role in modulating the boundary layer, suppressing turbulence, and maintaining flow attachment [9,10]. By inducing secondary flows or creating slip-like conditions, such surfaces alter the local shear stress distribution, thereby reducing drag [9,11]. Recent advancements in nanotechnology, surface chemistry, and precision manufacturing have enabled the realization of these complex textures on engineering components. Furthermore, these fine structures open opportunities for multifunctionality, including antifouling, noise reduction, and thermal regulation, positioning them as holistic design tools for next-generation aerospace and marine systems.

This review aims to systematically examine the state of the art of bio-inspired wing designs in aerodynamic and hydrodynamic enhancements. The current analysis attempts to provide an in-depth analysis of biological inspirations ranging from microstructured surfaces to dynamic morphing geometries. These surfaces are classified as static textures, superhydrophobic surfaces and adaptive smart structures. In addition, this paper delves into how biomimetic features influence flow behavior through vortex manipulation, transition delay, reconfiguration, wake stabilization and flow deceleration utilizing the findings from both experimental and computational studies. This paper also identifies current limitations such as scale-up challenges, integration difficulties, and sensitivity to varying flow conditions. Ultimately, this review outlines future research directions, emphasizing multifunctional designs, responsive materials, and standardized validation frameworks. It offers a comprehensive roadmap that bridges biological insights and fluid mechanics to inform the development of next-generation, high-performance aerodynamic and hydrodynamic systems.

## 2. Biological Inspirations for Aerodynamic and Hydrodynamic Performance Enhancements

Nature has evolved highly specialized surface structures that interact with fluid flow in remarkably efficient ways. These biological designs have evolved to minimize energy expenditure and enhance flow maneuverability, offering valuable lessons for engineering applications. This section explores several key biological inspirations and their mechanisms of drag reduction, emphasizing their structural characteristics, flow interaction principles, and their translation into biomimetic designs.

In the present study, biomimetic fine structures for aerodynamic and hydrodynamic enhancements are categorized into three principal types: (1) non-smooth static surfaces, (2) superhydrophobic and multiphase textures, and (3) dynamic or smart surfaces. These categories correspond to structural adaptations observed in aquatic and avian organisms, providing a design framework based on biological function and aerodynamic outcome.

### 2.1. Non-Smooth Static Surfaces

This category includes riblets, denticles and micro-grooves inspired by sharkskin and fish scales. These structures interact passively with turbulent flows by influencing the near-wall shear distribution and disrupting coherent structures such as streamwise vortices [16,17].

#### 2.1.1. Sharkskin Denticles

**Figure 2:**
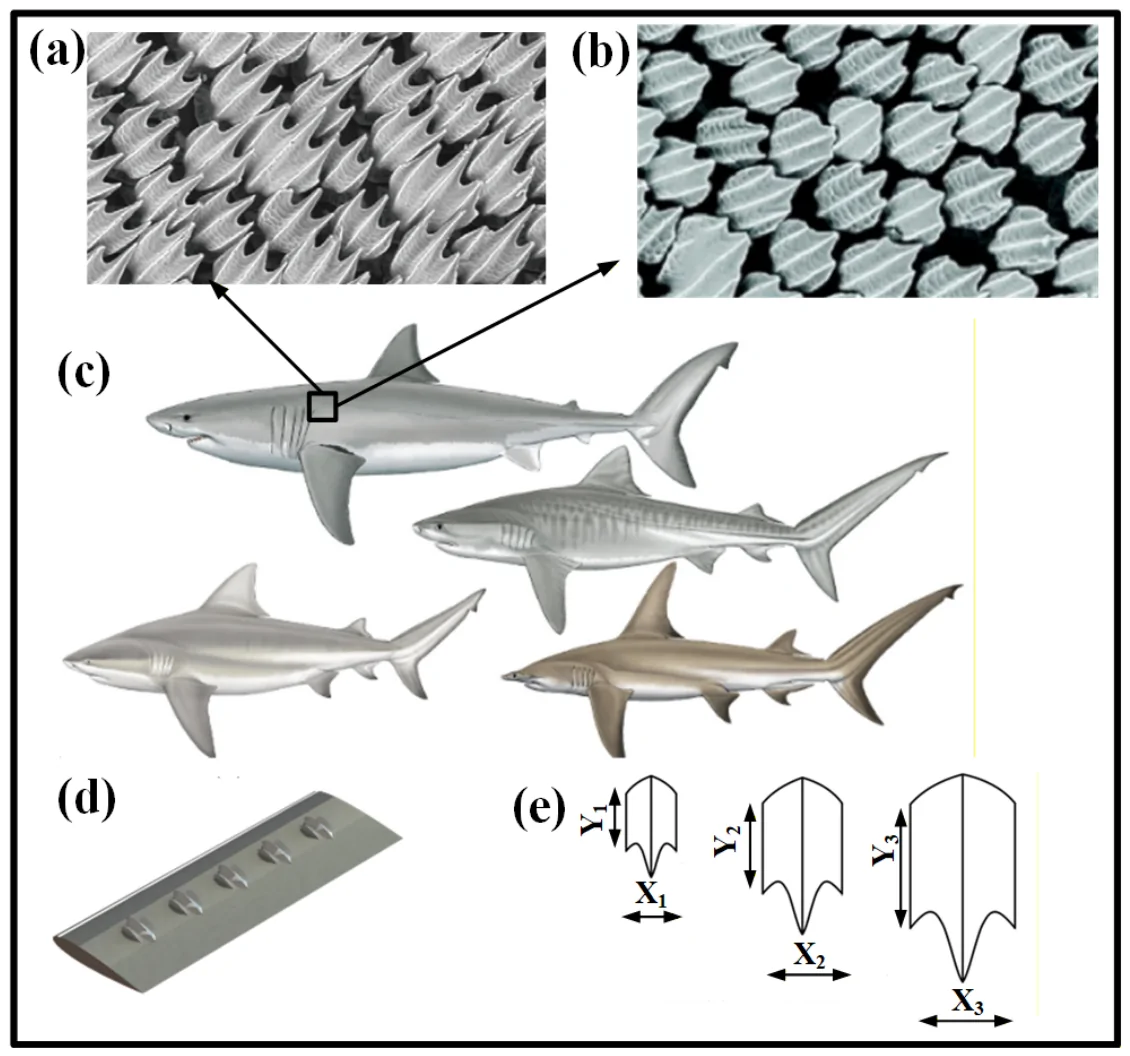
Sharkskin-inspired wing: (**a**) Riblets of sharkskin [21]. (**b**) Scanning Electron Microscopy (SEM) image of riblets of mako sharkskin [11]. (**c**) Sharks with different scales [22]. (**d**) Riblets on wing surface [23]. (**e**) Schematic representation of riblet dimensions [23].

Sharkskin, a widely studied biological surface, is composed of dermal denticles in microscopic, tooth-like scales aligned in the direction of local fluid flow [18,19]. These denticles contain riblet-like ridges that restrict streamwise vortices, reducing momentum loss in the boundary layer and suppressing turbulent bursts [20]. A schematic representation of sharkskin-inspired wing surface is presented in Figure 2. Sharkskin consists of different scale patterns (named riblets) usually found at different locations, as shown in Figure 2a,b. These riblet-like patterns have been widely considered for different aerodynamic studies. A typical arrangement of the riblets on the wing surface is depicted in Figure 2d. A schematic representation of the riblet dimensions is shown in Figure 2e. The obtained percentages of drag reduction from various studies are summarized in Table 1.

The fundamental drag reduction mechanism involves altering near-wall turbulent structures, effectively shifting the location of peak Reynolds shear stress away from the wall, which reduces the skin friction drag [30]. Dean and Bhushan [31] provided an extensive review of sharkskin drag reduction mechanisms, emphasizing how riblet geometry affects fluid mechanics in turbulent flows. They concluded that riblets can yield drag reductions of up to 10% under optimal conditions, primarily by mitigating cross-stream momentum transport. Domel et al. [25] extended this understanding by applying micro-computed tomography imaging to model individual denticles of the shortfin mako shark and 3D-printing those onto NACA0012 airfoils. Their results showed simultaneous increases in lift and reductions in drag at low angles of attack, with up to a 323% improvement in lift-to-drag ratio. The study revealed that denticles generate separation bubbles and streamwise vortices that enhance suction on the suction side of the airfoil while energizing the boundary layer, a dual mechanism rarely observed in traditional vortex generators. In addition, Martin and Bhushan [32] modeled various riblet shapes, including blade, scalloped, and scale-based geometries, and compared their performance. The blade geometry was found most effective, but their work also emphasized that drag reduction is sensitive to riblet dimensions and flow conditions, especially Reynolds number and wall-normal turbulence intensity. Furthermore, investigations have also integrated shark denticles into flexible and moving substrates. Wen et al. [21] developed flexible membranes with embedded synthetic denticles and tested them in robotic flapping motion, mimicking live swimming. Their experimental results indicated enhanced propulsion efficiency, up to 6.6% increased speed and 5.9% reduced energy cost at specific flapping amplitudes and frequencies, showing the promise of denticles on dynamic surfaces. Moreover, Hossain and Hyder [33] used CFD modeling in ANSYS 2022 R1 to simulate shark denticles on the upper surface of a NACA0012 wing. Employing the Shear Stress Transport (SST) k-ω turbulence model, their simulations demonstrated that shark denticles delayed boundary-layer separation and improved aerodynamic performance across multiple angles of attack. However, they also noted that optimal denticle spacing and inclination remain application-dependent and require further parametric study.

The experimental investigation conducted by Osman et al. [20] demonstrated that riblets with a height of 0.7 mm, a width of 2 mm, and a spacing equal to 0.75 times the riblet width achieved a maximum drag reduction of approximately 30%. Detailed flow-field measurements revealed that the superior performance of this geometry was associated with a pronounced suppression of Reynolds shear stress and a reduction in near-wall turbulence intensity. In contrast, a configuration with a riblet spacing equal to the riblet width exhibited a noticeable degradation in drag reduction capability, although it still operated within the transitionally rough regime. Furthermore, the remaining riblet configurations with different spacing investigated by Osman et al. [20] exhibited significant drag penalties under similar flow conditions even when the riblet height and width remain unchanged. Savino and Wu [34] investigated the drag reduction characteristics of a sharkskin-inspired surface and examined how specific denticle geometrical features influence the thrust-generating mechanisms. Their study revealed that beyond the conventional riblet-induced suppression of near-wall turbulence, the complex three-dimensional morphology of the denticles plays a crucial role in modifying the local flow field. In particular, they demonstrated that the unique riblet-like architecture promotes a penetrating reverse pore flow under adverse pressure gradients. This reverse flow generates localized thrust-producing effects while simultaneously preventing the occurrence of detrimental forward pore flow, which would otherwise increase flow resistance and result in a drag penalty. They reported a maximum drag reduction of approximately 4%, demonstrating that carefully designed sharkskin-inspired geometries can provide measurable hydrodynamic benefits through a combination of passive flow control and favorable pressure-driven flow interactions. Kang et al. [35] investigated the drag reduction of complex three-dimensional shark denticle-inspired surfaces, with particular emphasis on the influence of the underlying cavity structure and surface wettability. The authors reported that the superhydrophobic denticle surface achieved a maximum drag reduction of 19.91%, significantly outperforming its hydrophilic counterpart. This enhancement was attributed to the combined influence of the denticle geometry and the superhydrophobic coating, which together modify the fluid–surface interaction and reduce viscous resistance. Furthermore, Kang et al. [35] elucidated the underlying drag reduction mechanism as a synergistic interaction between the trapped air film and the vortical structures formed within the underlying cavities.

From the literature, it was noticed that sharkskin-inspired riblets constitute one of the most extensively investigated passive flow-control technologies for aerodynamic and hydrodynamic drag reduction [25]. Numerous experimental and numerical studies have reported drag reductions of up to 15%, demonstrating the significant potential of riblet surfaces for improving energy efficiency [20,24]. However, the effectiveness of riblets is highly sensitive to their geometric characteristics relative to the thickness of the viscous sublayer [32]. Most laboratory investigations are performed under carefully controlled turbulent-flow conditions, where the riblet geometry can be optimized for a specific Reynolds number and flow orientation [33]. Consequently, substantial drag reduction is generally achieved only within a narrow operational window. A relatively small increase in spacing weakened the riblet’s ability to suppress near-wall turbulence. Under such conditions, near-wall streamwise vortices penetrate into the riblet grooves, enhancing momentum exchange between the high-momentum outer flow and the low-momentum fluid adjacent to the wall [27]. Rather than suppressing turbulence production, the riblets then behave as roughness elements, leading to increased skin friction drag. In some cases, this may even exceed the drag levels observed over smooth surfaces. This sensitivity to geometric optimization partly explains the discrepancies reported in the literature [11,26]. Moreover, riblets are susceptible to fouling and mechanical wear, which can alter their micro-scale geometry and progressively degrade their drag reduction capability [11]. Consequently, the applicability of sharkskin riblets under realistic operational conditions remains an active area of research, particularly for full-scale aircraft and marine vehicles operating across a wide range of Reynolds numbers and environmental conditions.

#### 2.1.2. Fish Scales

**Figure 3:**
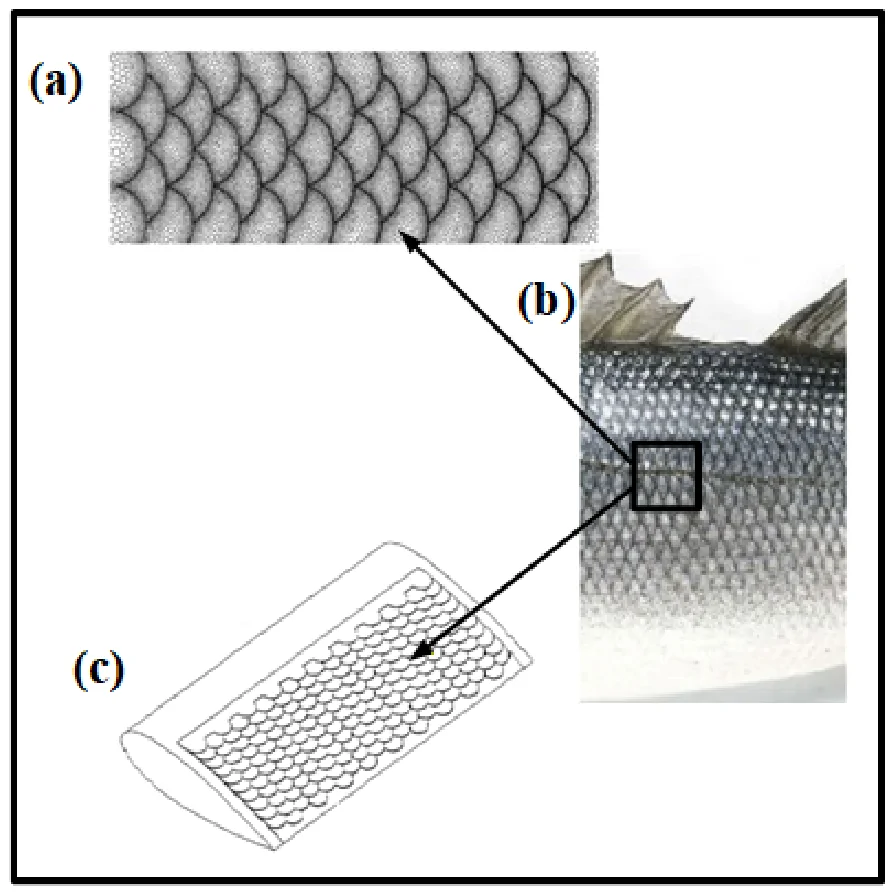
Fish scale-inspired wing: (**a**) Closer view of fish scales [39]. (**b**) Photograph of fish scales [40]. (**c**) Schematic representation of fish scales embedded on wing surface [38].

Fish scales present another compelling case of biological optimization for hydrodynamic and aerodynamic performance. Typically overlapped and covered with mucus, these structures reduce surface resistance by introducing micro-roughness and providing a lubricated interface that minimizes friction [36]. The mucus layer, in particular, reduces skin drag and prevents fouling, while the scale pattern modulates local flow dynamics [37]. The reported drag-reduction performance of different fish species is summarized in Table 2. Recent investigations, such as those conducted by Natarajan and Rose [38], utilized 3D-printed fish scale arrays (FSAs) mounted on NACA airfoil surfaces to assess their drag reduction capabilities under wind tunnel conditions. A schematic representation of the fish scales embedded on an NACA0021 wing surface is shown in Figure 3. The scale configurations, characterized by alternating ridge profiles and overlapping curvature, produced stable microvortices that delayed boundary-layer transition. Their experimental results revealed drag reductions approaching 10%, especially at low-to-moderate Reynolds numbers (Re), and highlighted the influence of scale angle and overlap ratio on vortex shedding and pressure recovery. However, optimal spacing and orientation remain sensitive to flow conditions and must be tuned for specific applications.

**Table 2:** Summary of fish scale-inspired drag reduction.

Morphology	Conditions	Mechanism	Drag Reduction
Tuna fish scales [41]	LES + Water tunnel at $Re=2.1\times 10^{5}$	Vortex stretching effect	7.22% (stress–strain sensor)
European bass [37,42]	$Re=33\times 10^{3}$	T-S wave attenuation	10–27% (numerical study)
Loach fish skin [43]	$Re=19.35\times 10^{4}$	Slippery mucous layer	9.5% (load cell)
Puffer fish skin [44]	Re = 44,554	Delay of transition	5–11% (pressure gradient-based measurements)
Ctenopharyngodon idelluse [45]	Flow rate of 0.66 m/s	Water trapping	3.014% (finite element analysis)
Jelly fish bell [46]	Water tunnel test	Mitigates VIV	—
Fish fins [47]	Flow velocity of 0.34 m/s to 0.96 m/s	Amplifies hydrodynamic loading	—
Fish mucous [48]	Viscosity ration = 0.8, Molecular stretch length = 80 and Weissenberg number = 20	Viscous and elastic theories	17% (numerical study)

Complementary computational studies using Reynolds-Averaged Navier–Stokes (RANS) modeling and SST k-ω turbulence models have shown that fish scale geometries induce secondary flow structures that suppress boundary-layer separation. These simulations support experimental findings by demonstrating improvements in lift-to-drag ratios and flow attachment in regions near the leading edge. For instance, Rong et al. [49] introduced a novel drag-reducing surface inspired by the anisotropic geometry and mucus-covered texture of bionic fish scales, termed lubricant-impregnated anisotropic slippery surface (LIASS). These surfaces were fabricated on aluminum–magnesium alloys using nanosecond laser scanning ablation to mimic periodic micro-/nanostructured arrays of fish scales. Thereafter, they were then impregnated with fluorinated lubricants to reduce surface energy and enhance slippage. The researchers employed both experimental and numerical methodologies. Experimentally, a self-assembled solid–liquid interface friction test setup was used to quantify drag forces. Additionally, anisotropic sliding and contact angles were measured to assess wettability. Numerically, a finite element model based on COMSOL Multiphysics v5.2a Multiphysics simulated drag behavior in laminar flow, highlighting the benefits of liquid–liquid interfacial slip. The key finding was that the LIASS exhibited direction-dependent drag reduction, with maximum reductions of 51.09% along span and 44.88% along chord at 2.9 m/s. This performance was attributed to the anisotropic alignment of micro-/nanostructures and the presence of a lubricant film that minimized viscous friction.

Unlike sharkskin, the exceptional hydrodynamic performance exhibited by many fish species cannot be attributed solely to the morphology of their scales. In natural aquatic environments, fish scales are typically covered by a thin mucus layer secreted by specialized epidermal cells [48]. This mucus layer serves multiple biological functions, including drag reduction, antifouling and lubrication. The synergistic interaction between the compliant mucus layer and the overlapping scale architecture is believed to contribute significantly to the remarkable swimming efficiency observed in many fish species [45]. However, replicating such a dynamic mucus-mediated lubrication mechanism in engineering applications remains extremely challenging. Maintaining a stable mucus-like layer on aerodynamic or hydrodynamic surfaces operating at high Reynolds numbers and elevated flow velocities is particularly difficult. Because the lubricating film can be rapidly depleted, sheared away, or destabilized by strong inertial forces and turbulent fluctuations [48]. Consequently, the majority of existing biomimetic studies have focused primarily on reproducing the geometric characteristics of fish scales, namely, fish scale overlapping arrangement, inclination angle, aspect ratio, and spacing, while largely neglecting the potential influence of the mucus layer [42]. This simplification may partly explain why the drag reduction capabilities reported for biomimetic fish scale surfaces are often substantially lower than those observed in living organisms.

A major source of inconsistency in the existing literature arises from the dual role played by fish scale topographies in modifying near-wall flow structures [37,42,49]. Several studies have demonstrated that appropriately designed fish scale arrays can delay laminar-to-turbulent transition by attenuating the growth of Tollmien–Schlichting waves [37,42]. This may generate favorable streamwise velocity streaks and modify the mean velocity profile in a manner that stabilizes the boundary layer. Under such conditions, the surface may effectively prolong laminar flow, thereby reducing overall skin friction drag. Conversely, other investigations have increased turbulent kinetic energy, leading to only marginal drag reduction associated with fish scale-inspired surfaces [41,45]. These contradictory observations suggest that fish scale textures can behave either as stabilizing or destabilizing elements depending upon flow conditions and geometric configuration employed. Parameters such as scale overlap ratio, scale height, inclination angle, spacing, edge sharpness, and orientation relative to the incoming flow strongly influence the resulting aerodynamic performance [37]. Even relatively small variations in these geometric parameters may alter the underlying flow mechanisms significantly, thereby leading to widely varying conclusions among different studies. In particular, excessively large scales or improperly oriented scale elements may induce localized flow separation, promote vortex shedding, and generate complex wake interactions in the near-wall region [41]. Instead of suppressing turbulence, such surface features may introduce additional disturbances into the boundary layer, thereby accelerating transition and increasing both pressure drag and skin friction drag. The sharp trailing edges of individual scales can act as distributed roughness elements that trigger the formation of separated shear layers and recirculation zones [49]. At higher Reynolds numbers, these separated flow structures may interact with one another, producing highly unsteady three-dimensional flow fields that negate any potential drag reduction benefits [41]. Furthermore, since the majority of fish species possess scales that are naturally aligned with the local flow direction during swimming, deviations from the optimal orientation in engineering applications may significantly degrade performance. It was noticed that the interaction between scale-induced vortices and naturally occurring coherent turbulent structures has received very limited attention. Similarly, the influence of fish scale textures on dynamic stall, flow reattachment, and vortex-dominated flows encountered in flapping wings and rotating blades has not yet been systematically investigated. Despite the growing interest in fish scale-inspired designs, their application to practical airfoils and lifting surfaces remains at a relatively early stage. Only a limited number of studies have evaluated the performance of fish scale arrays on actual airfoil geometries operating over a broad range of Reynolds numbers and angles of attack. Consequently, several important questions remain unanswered. It is still unclear whether the transition-delaying characteristics observed in idealized flat-plate studies can be retained in the presence of strong pressure gradients associated with cambered airfoils. Similarly, the influence of fish scale textures on lift generation, stall onset, aerodynamic hysteresis, and post-stall behavior has not been comprehensively explored.

### 2.2. Superhydrophobic and Multiphase Textures

**Figure 4:**
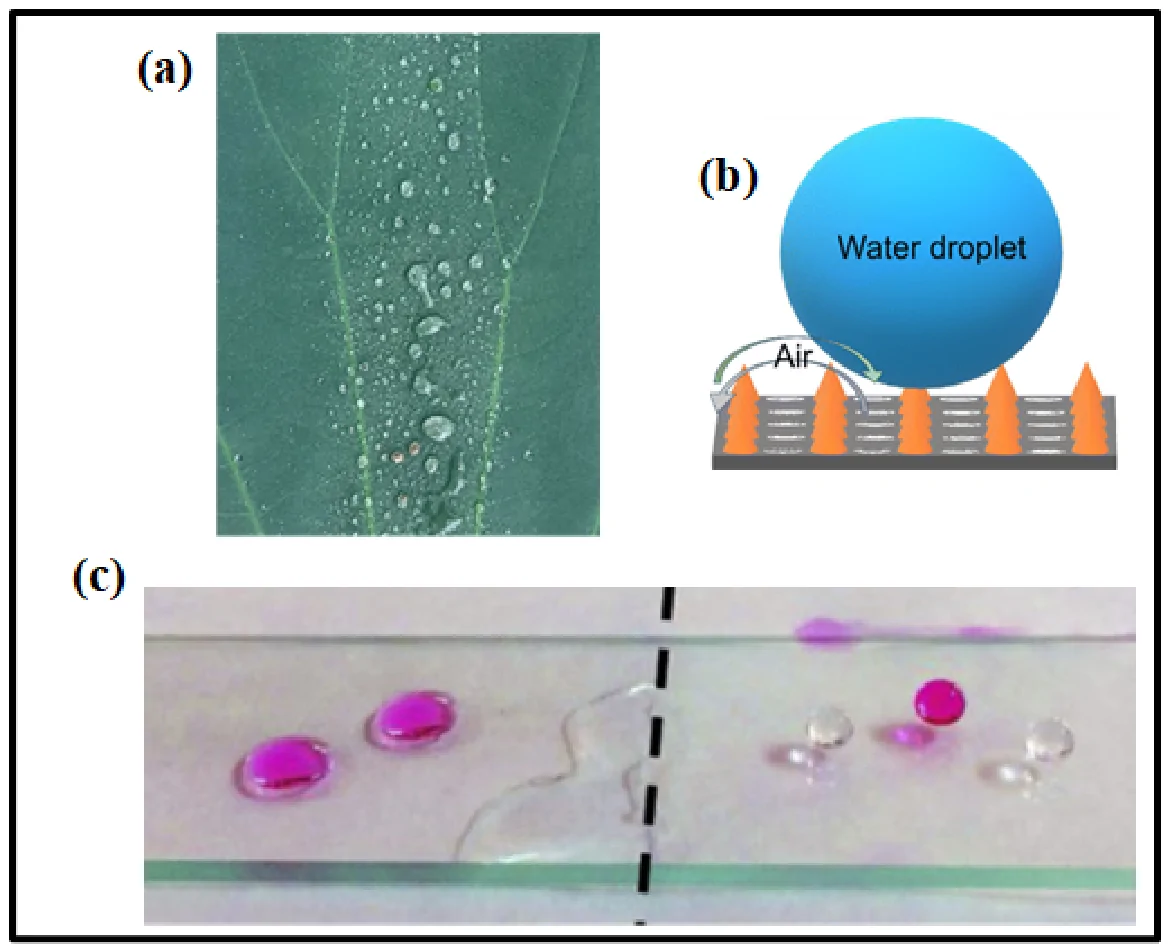
Superhydrophobic phenomena: (**a**) Lotus leaf with water droplets [59]. (**b**) Demonstration of superhydrophobic phenomena [59]. (**c**) Coated and non-coated surfaces [55].

Superhydrophobic surfaces (SHSs) are based on the Cassie–Baxter effect to trap air within surface cavities, forming a composite interface that reduces skin friction drag [50]. Inspired by lotus leaves and aquatic insects, these textures combine chemical hydrophobicity with hierarchical roughness. Numerous experimental validations such as water tunnel and pipe flow testing have confirmed drag reduction of up to 20%, with optimal parameters under laminar to transitional conditions [51]. Computational fluid dynamics (CFD) studies employ Volume of Fluid (VOF) and Large Eddy Simulation (LES) approaches to illustrate that slip length and gas layer stability are the primary governing factors [52]. The different superhydrophobic phenomena are listed in Table 3. The lotus leaf offers a prime example of a superhydrophobic natural surface, as shown in Figure 4. It exhibits dual-scale roughness: a combination of microscopic papillae and nanoscopic waxy projections that trap air and lead to high water repellency [53]. A schematic demonstration of this mechanism is shown in Figure 4b. This mechanism, known as the Cassie–Baxter effect, reduces contact angle hysteresis and has been adapted for drag reduction by minimizing the solid–liquid interface and allowing fluid to glide more easily across the surface. Experimental studies have demonstrated that superhydrophobic coatings inspired by lotus leaves can reduce frictional drag by up to 20% under laminar- and transitional-flow conditions [54]. A typical implementation of superhydrophobic coating with respect to the normal surface is shown in Figure 4c [55]. Recent studies have delved deeply into the potential of superhydrophobic surfaces, particularly those mimicking the lotus leaf as a passive strategy for drag reduction. The foundational work by Neinhuis and Barthlott [56] documented the water-repellent characteristics of over 200 plant species, including lotus leaves, which were found to possess hierarchical surface textures. This structural feature enables droplets to roll off the leaf surface, removing contaminants due to low adhesion and reduced contact area. Guo et al. [57] expanded on this by analyzing the mechanical architecture of the lotus leaf, identifying paralleled microtubes and layered hexagonal structures on the abaxial side that confer not only superhydrophobicity but also mechanical robustness. These observations are crucial to engineering durable bio-inspired coatings that do not compromise under mechanical stress. Manoharan and Bhattacharya [58] provided a broad review of superhydrophobic surface fabrication techniques, categorizing methods into top-down and bottom-up. Their work emphasized the importance of matching both the surface roughness and chemical functionality to replicate the Cassie–Baxter states. Their discussion of drag reduction noted that air entrapment at the interface reduces solid–liquid interaction, decreasing wall shear stress and enhancing flow.

**Table 3:** Summary of superhydrophobic surface configurations for drag reduction.

Morphology	Configuration	Mechanism	Drag Reduction
Superhydrophobic surface	Lotus leaf at $Re=4.7\times 10^{4}$ to $Re=2.35\times 10^{5}$ [60,61,62]	Frictional resistance suppression	5% to 77% (drag-measuring setup with dynamometer)
	Rice leaf at $Re=12.5\times 10^{3}$ [63]	Anisotropic flow	26% (pressure drop measurements)
	Water strider [64]	Non-wetting surface	—
	Butterfly wing [65]	Anisotropic flow	29% (pressure drop measurements)

Numerous experimental and numerical investigations have reported substantial reductions in skin friction drag, with some laboratory studies demonstrating drag reductions as high as 80% under carefully controlled conditions [60]. These remarkable improvements are primarily attributed to the formation of a stable air layer. The air gets trapped within the hierarchical micro- and nanostructures present on the surface. These entrapped air pockets minimize direct contact between the fluid and the solid substrate, thereby reducing viscous shear stresses and facilitating partial slip at the wall [51]. However, despite these encouraging findings, significant discrepancies exist among reported studies. The most critical factor governing the effectiveness of SHSs is the stability of the trapped air layer. Under ideal laboratory conditions, the surface typically remains in the Cassie–Baxter state, wherein the fluid rests on the tips of the microstructures while air remains trapped within the surface cavities. In this state, the effective contact area between the liquid and the solid is significantly reduced, resulting in lower skin friction drag. However, under realistic aerodynamic and hydrodynamic operating conditions, maintaining a stable Cassie–Baxter state becomes increasingly challenging. Elevated Reynolds numbers, high dynamic pressures, prolonged immersion, intense turbulent fluctuations, cavitation, and adverse pressure gradients can destabilize the entrapped air layer [51]. Once the local pressure exceeds a critical threshold, the air pockets collapse, and the liquid penetrates into the surface cavities, causing a transition to the fully wetted Wenzel state [53]. In the Wenzel state, the fluid completely fills the microstructures, substantially increasing the solid–liquid contact area. Consequently, the slip effect disappears, and the surface may behave as a conventional rough surface, often resulting in increased skin friction and pressure drag.

The underlying flow physics associated with air pocket degradation are also complex and not yet fully understood. In turbulent flows, coherent vortical structures continuously interact with the air–water interface trapped within the surface texture [60]. Strong turbulent bursts may deform the gas–liquid interface, induce interface oscillations, and promote localized wetting. Similarly, in cavitating flows, vapor bubble collapse near the surface can generate intense pressure pulses capable of destroying the microstructures and destabilizing the trapped air layer [64,66]. In aerodynamic applications, the interaction between high-speed flow, surface vibrations, and compressibility effects may further complicate the maintenance of a stable air layer [66]. Therefore, the drag reduction performance of SHSs is often highly transient, with significant temporal variations depending on the evolution of the gas layer. Another major challenge limiting the practical deployment of SHSs is the long-term durability of the coatings. The micro- and nano-structures responsible for superhydrophobicity are inherently fragile and highly susceptible to mechanical and environmental degradation [53]. Even minor alterations in surface morphology may significantly compromise air layer stability and consequently diminish drag reduction performance. Since aircraft surfaces are routinely exposed to harsh environmental conditions over extended operational periods, preserving superhydrophobic functionality throughout the service life remains a formidable engineering challenge [51]. Similarly, marine applications introduce additional complexities. Prolonged exposure to seawater may lead to salt deposition, mineral scaling, and biofouling [51]. Such fouling not only modifies the intended surface topography but also promotes the premature wetting of the surface cavities, thereby destabilizing the air layer. Furthermore, increasing hydrostatic pressure with immersion depth compresses the trapped air pockets and accelerates the Cassie-to-Wenzel transition [62]. Consequently, although SHSs have demonstrated exceptional drag reduction capabilities in laboratory environments, sustaining these benefits under long-term submerged conditions remains difficult. A further limitation arises from the scale at which most investigations have been performed. The majority of experimental studies are conducted using relatively small specimens under highly idealized flow conditions. Such conditions do not accurately represent the complex operational environments encountered by full-scale aircraft and marine vessels. Scaling SHSs from laboratory specimens to large engineering structures while maintaining uniform surface morphology, consistent air layer stability and long-term durability remains an unresolved challenge.

### 2.3. Dynamic and Smart Surfaces

Smart surfaces inspired by compliant skin of dolphins and flexible filaments adapt to changing flow regimes using passive deformation or active actuation [67]. Vegetation-inspired canopy exemplifies this group [68]. These surfaces change topology or stiffness in response to external stimuli such as pressure fluctuation, shear stress, or gust loading [69].

#### 2.3.1. Compliant Dolphin Skin

The skin of cetaceans, particularly that of dolphins, exemplifies dynamic compliance, a property that significantly contributes to reducing drag during high-speed swimming [70]. This skin comprises multiple layers with varying elastic moduli, enabling anisotropic mechanical behavior. It passively responds to flow-induced instabilities by absorbing energy from vortical structures and suppressing their amplification within the boundary layer [71]. This mechanism allows dolphins to mitigate Vortex-Induced Vibrations (VIVs) and delay the transition from laminar to turbulent flow, enhancing hydrodynamic efficiency [71]. The summary of different dolphin-inspired compliant surfaces and the respective quantitative values of drag reduction percentages are presented in Table 4.

Compliant skin dynamically adjusts to pressure fluctuations by deforming in response to external forces, thus modifying the wall shear stress distribution and stabilizing the boundary layer [72]. This effect has been modeled using linear stability theory and direct numerical simulation (DNS) [72,73]. It was reported that the skin can suppress Tollmien–Schlichting wave growth [72]. Computational studies, including the finite element modeling of viscoelastic layers, have explored the influence of skin thickness, stiffness gradients, and damping coefficients on flow stability and surface drag. Experimental replication efforts have included the development of synthetic compliant membranes composed of viscoelastic polymers embedded with fiber reinforcements. These artificial skins have been applied to flat plates and airfoils and then tested in water channels and wind tunnels to evaluate their performance. The results show that optimized skins can achieve drag reduction of up to 7–9% under specific flow regimes [72].

**Table 4:** Summary of dolphin-inspired compliant surface configurations for drag reduction.

Morphology	Configuration	Mechanism	Drag Reduction
Compliant dolphin skin	15 mm thick silicone rubber compliant coating	Enhancement in viscous sublayer thickness	7% (floating drag balance measurement) [74]
	2 mm thick silica-based flexible coating	Fluid damping suppression	12% (drag measured through pressure losses) [75]
	Compliant n-octadecane film	Slip velocity	49% (drag measured using dynamometer) [76]
	Black color coating	Heated boundary layer	7% (drag measured through thermal analysis) [77]
	Silicone polymer-based compliant coating	Stabilization of laminar boundary layer	3% (force balance with load cell) [78]
	PDMS/ethyl acetate-based flexible coating	Suppression of frictional resistance of wall	21.6% (drag force device equipped with torque sensors) [79]

Zheng et al. [71] investigated a dolphin-inspired transverse-wave micro-groove surface (TWMS), which was engineered with sinusoidal transverse grooves intended to disrupt turbulent structures and reduce skin friction drag. The research study employed a dual approach: numerical simulations using the SST *k*-ω turbulence model and experimental validation in a closed water tunnel. It was reported that the TWMS with moderate groove amplitude-to-wavelength ratios achieved drag reduction of up to 8.74%, outperforming both smooth and conventional riblet surfaces under identical flow conditions. The transverse grooves induced spanwise vortical disturbances that effectively thickened the viscous sublayer, weakened sweep and burst cycles, and stabilized low-speed streaks near the wall, contributing to reduced turbulent momentum exchange.

**Figure 5:**
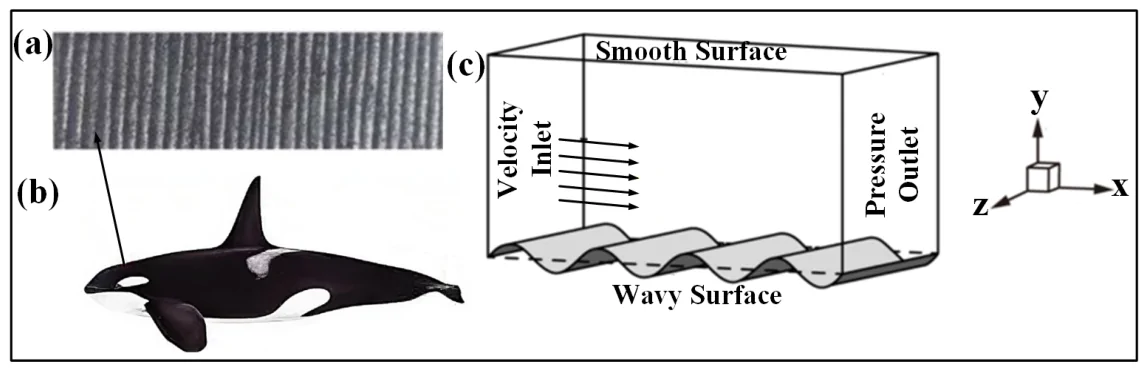
Schematic representation of dolphin-inspired oscillating surface. (**a**) Photograph of dolphin skin [71]. (**b**) Depiction of dolphin. (**c**) Computational domain with wavy boundary [80].

Wang and Liu [70] proposed an aerodynamic drag reduction technique that draws from the microvibrational behavior of dolphin skin, specifically mimicking the localized transverse-wave motion observed in live dolphins. Their experimental setup involved a flat-plate model outfitted with ultrasonic piezoelectric actuators to induce high-frequency (20–40 kHz), low-amplitude (2–8 µm) surface vibrations under controlled subsonic wind tunnel conditions. A schematic representation of a dolphin skin-inspired surface is shown in Figure 5. Moreover, URANS-based CFD simulations were conducted to characterize turbulence modulation and wall shear stress behavior. The authors demonstrated that the ultrasonic microvibrations significantly alter the turbulent boundary layer by disrupting coherent vortical structures and turbulent bursts. This resulted in drag reduction of up to 13.7% under optimal actuation (30 kHz, 6 μm). These vibrational effects promoted the formation of a thicker viscous sublayer and delayed near-wall turbulence regeneration. The PIV images revealed a noticeable suppression of streak formation, while wall-pressure fluctuation data confirmed reduced turbulent intensity. Drag reduction could be achieved without altering the geometry of the surface, which provides adaptability and functiona simplicity.

Compliant dolphin skin has long been hypothesized to suppress turbulence through passive interactions between the deformable wall and near-wall coherent turbulent structures. The concept originated from observations that dolphins exhibit exceptionally high swimming efficiency, which led researchers to speculate that the viscoelastic properties of dolphin skin may actively influence the structure of the surrounding boundary layer [80]. Early experimental investigations reported substantial drag reductions, in some cases exceeding 20%, thereby stimulating extensive research into compliant coatings as passive flow-control devices [79]. However, subsequent experimental and numerical studies have produced highly inconsistent results, leading to considerable controversy regarding both the magnitude of achievable drag reduction and the underlying physical mechanisms responsible for the observed performance [73,81]. The fundamental mechanism by which compliant surfaces are expected to reduce drag is based on their ability to interact dynamically with near-wall flow structures. In turbulent boundary layers, coherent structures such as low-speed streaks, quasi-streamwise vortices, and bursting events are primarily responsible for the production and transport of turbulent kinetic energy [81]. An appropriately designed compliant surface can deform in response to pressure fluctuations induced by these coherent structures. If the wall deformation occurs with suitable amplitude and phase relative to the turbulent fluctuations, a portion of the turbulent energy can be absorbed by the compliant layer and dissipated through material damping [80]. Consequently, the intensity of near-wall turbulent events may be attenuated, leading to a reduction in Reynolds shear stress and a suppression of turbulent momentum transfer toward the wall. In addition, wall deformation may alter the spatial organization of coherent structures, weaken vortex–wall interactions, and reduce the frequency of bursting events, all of which contribute to lower skin friction drag.

Despite these promising mechanisms, studies conducted during the late 2010s and early 2020s increasingly converged toward the conclusion that compliant coatings are most effective under laminar- and transitional-flow conditions [73,74,75,80]. In such regimes, the dominant flow instabilities are relatively well-organized and characterized by narrow frequency bands. This allows the compliant surface to interact effectively with instability waves, such as Tollmien–Schlichting waves, and delay the onset of transition [80]. The deformable wall can absorb energy from these instability modes, thereby suppressing their growth and extending the laminar-flow region. However, in fully turbulent flows, the boundary layer contains a broad spectrum of interacting scales and frequencies. This can be significantly more difficult for a passive compliant surface to simultaneously suppress all energetically important turbulent motions [73]. Consequently, there remains considerable debate regarding whether compliant coatings can produce meaningful drag reduction in high-Reynolds-number turbulent flows relevant to practical aerospace and marine applications [81]. One of the principal challenges associated with compliant coatings is their extremely narrow operational window. Effective drag reduction is achieved only when the mechanical properties of the coating are carefully tuned to match the characteristic frequencies and wavelengths of the dominant near-wall turbulent structures [75]. If the coating is excessively stiff, the wall behaves almost as a rigid surface, thereby eliminating any beneficial fluid–structure interaction. Conversely, if the coating is too soft, excessive wall deformation may occur, resulting in amplification rather than suppression of flow instabilities. Under such conditions, the compliant wall may undergo resonance with turbulent pressure fluctuations [73]. This leads to the generation of traveling-wave flutter, hydroelastic instabilities, or self-sustained oscillations. Instead of reducing drag, these mechanisms may enhance turbulent production, increase Reynolds stresses, and ultimately cause drag penalties [81]. In extreme cases, large-amplitude oscillations can trigger additional flow separation, significantly deteriorating aerodynamic or hydrodynamic performance.

Discrepancies among published studies are particularly pronounced. Reported drag reductions range from negligible values to more than 20%, while several investigations have observed substantial drag increases [76,81]. These inconsistencies arise because compliant-wall behavior is strongly influenced by numerous interdependent parameters. Small variations in these parameters can alter the fluid–structure interaction mechanisms considerably, leading to entirely different outcomes. Another important limitation is that most existing compliant-wall studies employ simplified homogeneous and isotropic materials [73]. Materials such as silicone rubbers or elastomeric coatings do not accurately replicate the complex biological architecture of natural dolphin skin [78]. Real dolphin skin is a highly sophisticated multilayered structure consisting of anisotropic collagen fibers, compliant connective tissues, and viscoelastic subdermal layers [81]. This hierarchical organization enables the skin to exhibit spatially varying stiffness, directional mechanical properties, and adaptive deformation characteristics. Such anisotropic and multilayered behavior is difficult to reproduce using conventional engineering materials [76]. Consequently, the performance of synthetic compliant coatings may differ substantially from that of natural dolphin skin, raising important questions regarding the validity of direct biological analogies.

#### 2.3.2. Flexible Filaments and Vegetation Canopy

**Figure 6:**
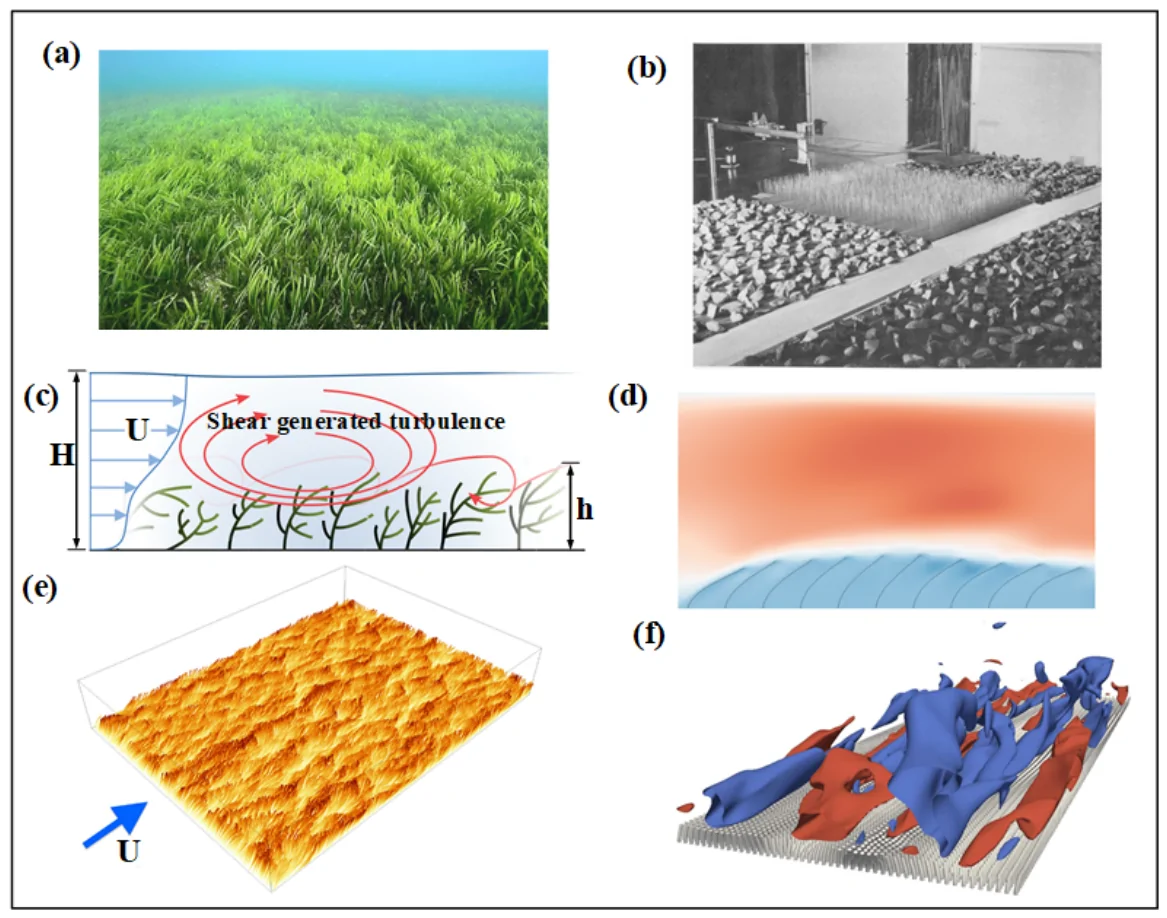
Channel flow with flexible filaments: (**a**) Submerged dense vegetation canopy [13]. (**b**) Wind tunnel setup of vegetation canopy [83]. (**c**) Oscillatory flow around vegetation canopy [13]. (**d**) Flow over a row of flexible filaments [84]. (**e**) Coherent wave motion of flexible canopy [85]. (**f**) Streamwise velocity flow over flexible filaments [86]. (**H**: channel height; **h**: filament height).

The quest for energy-efficient flow management has driven intensive research into passive flow-control strategies that require no external actuation yet yield substantial reductions in drag. Among these, flexible filaments and vegetation-inspired canopies, as shown in Figure 6, have attracted growing attention for their ability to self-adapt to local fluid forces, thereby harnessing fluid–structure interactions to optimize performance. Unlike rigid vortex generators or fixed-geometry roughness, flexible filaments reconfigure their shape in response to varying flow conditions (see Figure 6), stabilize near-wake vortices, decelerate boundary-layer momentum transfer, and dynamically interact with shear-layer instabilities. These mechanisms operate synergistically to reduce both form and friction drag by up to 30% in laminar and transitional regimes while enhancing mixing and delaying separation. Different reported numerical/experimental studies of flexible filaments and vegetation canopy in flow with key conditions and outcomes are presented in Table 5. In early small-scale experiments, researchers investigated single flexible filaments clamped at one or both ends and subjected to uniform or parabolic channel flow. These setups, typically acrylic recirculating flumes with L>>D, allowed for precise control of the Reynolds number (Re ∼ $10^{2}$–$10^{3}$). The experimental studies mostly used silicon or rubber filaments whose stiffness (Young’s modulus *E* ∼ 1–2 MPa) could be measured and varied. Flow-induced filament deformation and onset of flapping were documented using high-speed cameras (up to 10,000 fps), with tip deflection and angular amplitude being tracked via direct image analysis or digital image correlation. For instance, Zhang et al. [82] experimentally observed a clear transition from static deflection to steady flapping as flow speed increases. It was reported that the findings were consistent with theoretical predictions based on the filament’s Cauchy number (i.e., $Ca=\rho U_{\infty }^{2}L^{3}/EI$). Furthermore, the documented vortical shedding in the wake synchronized with filament motion in the lock-in regime.

Subsequent experiments placed doubly clamped filaments across channel spans to explore flapping under cross-flow constraints. In these larger setups, edge-fixed filaments spanning the channel underwent richer dynamics, including standing waves, traveling waves, and chaotic beating as flow speed increased. Zhang et al. [87] documented five regimes, namely, static bent, small oscillations, standing wave, traveling wave, and chaotic flapping, by systematically varying filament length and inclination. Wake visualization via dye injection revealed coherent vortex rings shed from traveling modes. This intricately modified local pressure field observations, later supported by IBM–DNS simulations showing similar pressure redistribution enhancing lift-to-drag ratios.

Further explorations involved wall-mounted filament arrays, where hundreds or thousands of slender flexible stems attached to the floor of the channel simulate hairy surfaces or vegetation beds. Singh et al. [68] spaced 5–10 cm long, low-stiffness filaments at two-diameter intervals across a channel floor and measured mixing via tracer dispersion and velocity statistics using PIV and hot-film shear sensors. They found optimal flexibility (tuned stiffness), where mixing improved by 25–40% without significant drag increase (<5%), balancing momentum transfer and minimal blockage. In a variation, randomly spaced arrays disrupted wake coherence and improved drag reduction (∼8–10%) while enhancing mixing, demonstrating that filament spacing and stiffness interplay affects macroscopic fluid dynamics.

**Table 5:** Numerical/eperimental studies of flexible filaments or vegetation canopy in flow with key conditions and outcomes.

References	Configuration	Re Range	Finding
Kunze & Brucker [88]	Cylinder with self-adaptive flaps	Re ∼ $10^{4}$	Wake realigns and shedding jumps
Favier et al. [89]	Oscillating channel with 10 wall flaps	Re ∼ 120	Honami waves and reduced recirculation
Mao et al. [90]	Stationary cylinder + flapping filament	Re ∼ 150	Clapping filaments outperform snaking
Hasegawa et al. [91]	Smooth vs. microfiber-coated cylinder	Re ∼ $2\times 10^{4}–10^{5}$	Delay of transition and 32% drag reduction
Afrouzi et al. [92]	Laminar channel, multiple wall filaments	Re<<100	Flow stabilization and suppression of large vortex formation
Xiong et al. [93]	Oscillatory boundary layer over wall filaments	Re ∼ $10^{2}$	Identified undulation modes and effective slip
Davoudian et al. [94]	Cylinder + flexible micro-tail	Re ∼ 10	Longer tail leads to drag reduction
Xu et al. [84]	Free flexible flap	$Re\in [0.1,10^{2}]$	Morphological evolution of the flap
Li et al. [95]	2D thin, flexible plate in turbulent flow	Unspecified turbulent flow	Up to 12.99% drag reduction
Wang et al. [96]	Flexible vegetation canopy in open channel	Re ∼ $2\times 10^{4}–5\times 10^{4}$	Exhibits coherent monami waves
Wang et al. [97]	Submerged flexible vegetation in open channel	Re ∼ $2\times 10^{4}–5\times 10^{4}$	$C_{d}$ drops from 10.7 to 2.11 as Re goes from $2\times 10^{4}$ to $5\times 10^{4}$
Rota et al. [98]	Dense fully submerged canopy	Re ∼ 5000	Prevents vertical exchange of momentum
Rota et al. [99]	Single wall-mounted flexible fiber	Wall turbulence; Re unspecified	Characterized flapping regimes
Tang et al. [100]	Submerged flexible canopy (3 densities)	Turbulent flow; Re not specified	Highlights that flexible plants increase flow resistance
Tang et al. [101]	Submerged flexible canopy (2 densities)	Low-to-moderate flow	Illustrates how reconfiguration affects drag and mixing
Zhang et al. [87]	Flexible filament with both ends fixed	Low-Re regime	Identifies multiple motion modes
Munoz et al. [102]	Filaments attached to D-shaped bluff body	Moderate Re; laminar/turbulent transition	Passive deflection reduces wake recirculation
Wu et al. [103]	Submerged vegetation with varying stiffness	Re unspecified; lab scale	Stronger reconfiguration yields smaller $C_{d}$
Dang et al. [104]	Submerged vegetation canopy	Low Re	Observed transition from swaying to monami
Chen and Li [105]	Single wall-mounted stem	Re ∼ 200–1000	Identifies three vortex-shedding regimes as Re grows
Kim et al. [106]	Submerged vegetation canopy	Low Re; laminar steady flow	Identifies five flow regimes
Deng et al. [107]	Clamped flexible membrane	Turbulent flow	Identifies three flutter modes
He et al. [108]	Explicit flexible-stem canopy flow	Turbulent-flow regime	Waving input can be up to ∼50% of shear production near canopy top

Despite considerable attention received over the past decade, the aerodynamic and hydrodynamic performance of flexible filament coatings remains far from fully understood. One of the most significant challenges arises from the existence of multiple flow regimes resulting from the complex fluid–structure interactions between the flexible elements and the surrounding flow [87,106]. Depending on the Reynolds number, Cauchy number, aspect ratio and spacing, filament arrays may exhibit a wide variety of dynamic behaviors. This may include small-amplitude oscillations, synchronized waving, asynchronous motion, collective reconfiguration, large-amplitude flapping, and even chaotic oscillations [108]. These distinct dynamical states can profoundly influence the near-wall flow structures and, consequently, the overall drag characteristics. Moderate and coherent filament oscillations may generate favorable flow modifications by disrupting energetic large-scale turbulent structures and redistributing turbulent kinetic energy [98]. In dense canopies, the filaments may also undergo flow-induced reconfiguration, whereby the flexible elements bend in the streamwise direction, effectively reducing their frontal area and minimizing form drag [105]. Furthermore, coherent waving motions analogous to the monami phenomenon observed in aquatic vegetation can produce a shear layer above the canopy. This may partially isolate the wall from the outer turbulent flow, thereby reducing momentum exchange and suppressing large-scale vortical motions.

However, these beneficial mechanisms are not universally observed. Excessive filament deformation may produce entirely different flow responses. When the structural stiffness is insufficient or the flow velocity becomes excessively high, large-amplitude flapping motions can develop, resulting in enhanced turbulent mixing, increased vortex shedding, and elevated drag [109]. In particular, asynchronous or chaotic filament oscillations may inject additional disturbances into the flow, intensify turbulence production, and promote momentum transfer toward the wall [99]. Under such conditions, the filament layer effectively behaves as a rough, highly unsteady surface rather than a drag-reducing canopy. These discrepancies highlight the existence of a narrow parameter space within which flexible filament coatings can operate effectively. Identifying the optimal combinations of filament stiffness, length, spacing, and arrangement therefore remains a major research challenge [108]. Long-term structural durability represents another critical limitation hindering the widespread implementation of flexible filament technologies. Since the filaments are continuously subjected to oscillatory hydrodynamic or aerodynamic loading, repeated deformation cycles may induce material fatigue, creep, fracture, or permanent deformation [96]. Over extended operational periods, such degradation may alter the effective stiffness and dynamic response of the filaments, thereby significantly modifying the intended flow-control mechanisms. From a computational perspective, accurately predicting the behavior of flexible filament arrays remains an extremely demanding task. The flow around flexible canopies involves strong two-way fluid–structure interactions, large structural deformations, and highly unsteady turbulent-flow phenomena occurring across a broad range of spatial and temporal scales [93,108]. Capturing these interactions requires fully coupled fluid–structure interaction (FSI) simulations. This often involves high-fidelity numerical approaches such as Direct Numerical Simulation (DNS), Large-Eddy Simulation (LES), or Immersed Boundary Methods (IBMs) coupled with nonlinear structural solvers [85]. Such simulations are computationally expensive, particularly when considering realistic filament densities and high-Reynolds-number flows. As a result, many existing numerical studies rely on simplified assumptions, including reduced-order structural models, linear elasticity approximations, or idealized flow conditions. Although these simplifications provide valuable physical insights, they may not adequately capture the complex dynamics observed in practical applications.

## 3. Mechanisms of Aerodynamic and Hydrodynamic Performance Enhancements

**Figure 7:**
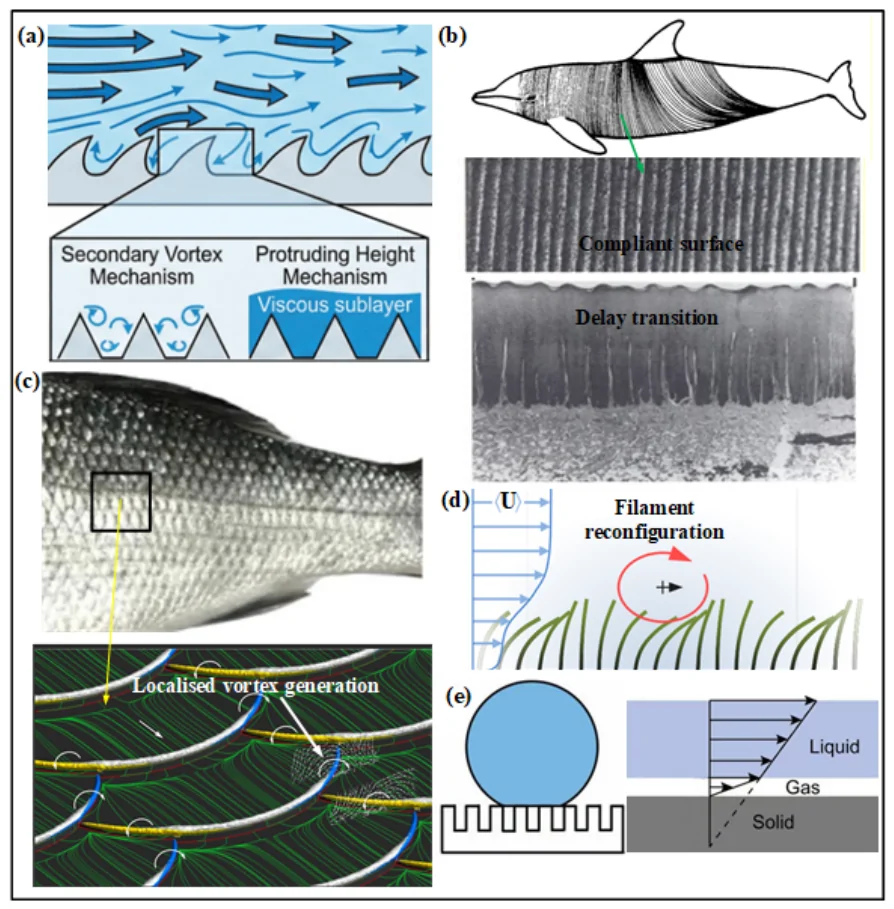
Bio-inspired mechanisms for drag reduction: (**a**) Sharkskin-inspired riblets modifying near-wall vortices and viscous sublayer characteristics [112]. (**b**) Compliant surface of dolphin skin delaying transition [113]. (**c**) Fish scale-inspired vortex generation mechanism [39]. (**d**) Reconfiguration of filament shape modifying tip vortices [13]. (**e**) Superhydrophobic surface slip mechanism [114].

Understanding the mechanisms through which bio-inspired surface and morphological structures enhance aerodynamic/hydrodynamic characteristics is essential to optimizing their application in engineering systems. Table 6 provides a comparative summary of the bio-inspired surfaces discussed in this section, highlighting their biological inspiration, underlying flow-control mechanisms with reported maximum possible drag reduction. Each mechanism differs on the basis of the structural category and the nature of fluid interaction. However, most converge on the principles of boundary-layer control, vortex suppression, laminar-flow retention and flow reattachment. A schematic illustration of different bio-inspired mechanisms is depicted in Figure 7. Although significant drag reduction and flow-control capabilities have been achieved under controlled laboratory conditions, each bio-inspired surface exhibits distinct physical constraints and practical challenges that limit large-scale implementation.

### 3.1. Vortex Manipulation

**Figure 8:**
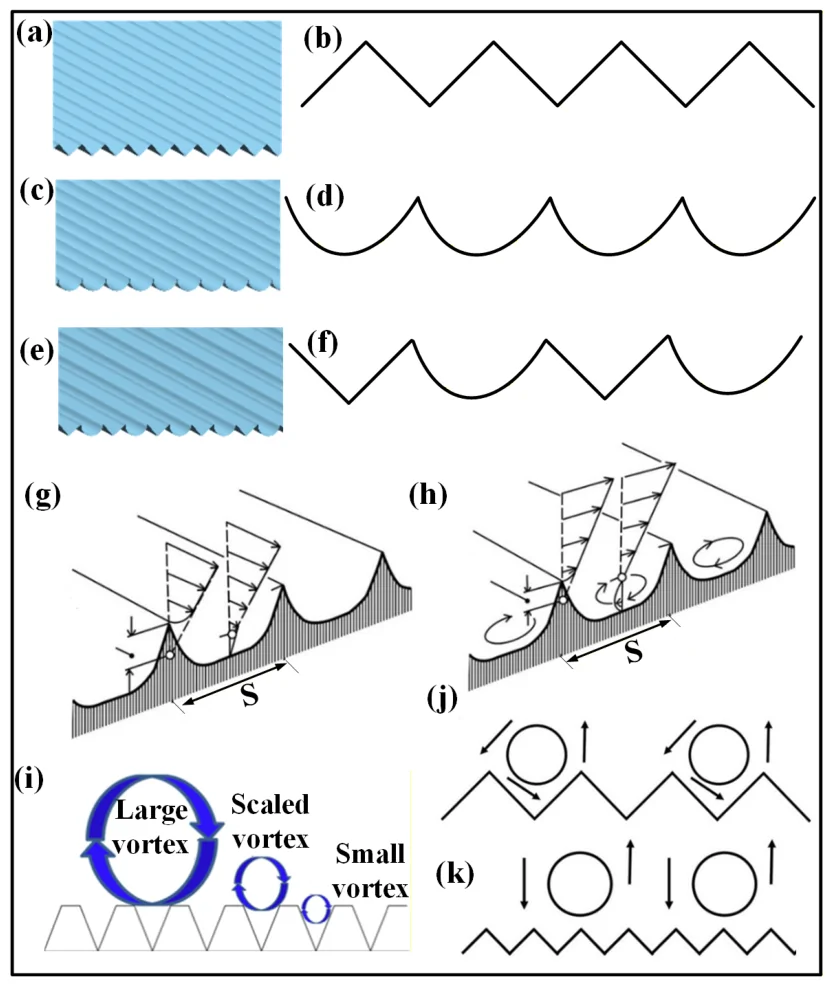
Sharkskin riblet-inspired drag reduction characteristics: (**a**,**b**) Sawtooth configuration [24]. (**c**,**d**) Scalloped configuration [24]. (**e**,**f**) Hybrid configuration [24]. (**g**) Longitudinal protrusion height [117]. (**h**) Cross-flow protrusion height [117]. (**i**) Streamwise vortical structures [118]. (**j**) Schematic vortical rotation for drag increment [119]. (**k**) Vortex lift for drag reduction [11] (**s**: center-to-center distance between riblets).

**Figure 9:**
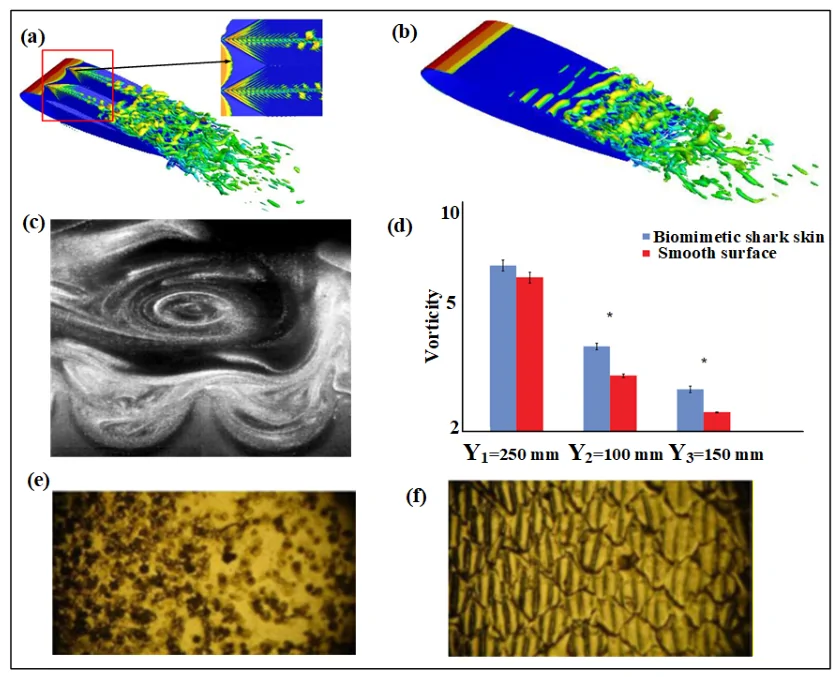
Variation in instantaneous velocity around the wing: (**a**) Wing with riblets. (**b**) Without riblets [24]. (**c**) Flow along the wing’s cross-section in the form of streamlined vortices [116]. (**d**) Maximum vorticity at three different motions [21]. (**e**) Fouling of smooth surface. (**f**) Antifouling of sharkskin-inspired biomimetic surface [120].

One of the most studied mechanisms is vortex manipulation through surface patterning. Sharkskin riblets can create streamwise grooves that can stabilize turbulent boundary layers by generating quasi-streamwise vortices [115]. Different riblet patterns and their influence on vortex evolution is schematically demonstrated in Figure 8. These microvortices align with the main flow and disrupt the formation of large-scale turbulent eddies to delay flow separation. To verify this, Hijazi and Tolouei [24] conducted Large Eddy Simulations (LESs) on wings with and without riblets. The findings indicate that the wing with riblet pattern functions as an effective vortex generator. The vortical structures originate from the tips of the diverging sections and coalesce along the airfoil, as shown in Figure 9. Thereafter, these vortices reach convergence lines that enable the boundary-layer airflow to resist adverse pressure gradients for a longer duration. This behavior contrasts with that observed on wing surface without riblets, where vortices typically originate approximately at the mid-chord region, as shown in Figure 9b. Furthermore, these riblets initiate vortex formation earlier than conventional wings, specifically at the diverging-region tips. This further enhances the delay of flow separation, as shown in Figure 9. Lee and Lee [116] confirmed this effect through PIV-based visualizations, showing that well-aligned riblets displace high-shear zones further from the surface, leading to reduced turbulent momentum exchange, as presented in Figure 9c.

Wen et al. [21] conducted a comparative study analyzing the maximum vorticity fields over a 3D-printed sharkskin-inspired wing surface and a conventional, smooth wing surface, focusing on their influence on the formation of leading edge vortex (LEV). Their results revealed that the sharkskin-inspired wing surface significantly intensified the LEV compared with the conventional, smooth wing. Specifically, the riblet-like features of the sharkskin promoted earlier and stronger vortex roll-up near the leading edge, resulting in higher peak vorticity magnitudes, as shown in Figure 9d. This enhanced vortex strength is indicative of improved flow attachment and increased lift generation, particularly beneficial during high-angle-of-attack flight regimes. Furthermore, these riblets were hypothesized to act as passive vortex promoters, stabilizing the LEV and delaying flow separation. These findings demonstrate the aerodynamic potential of sharkskin-inspired textures not only for drag reduction, as traditionally explored, but also for augmenting lift through improved vortex dynamics. Domel et al. [25] expanded on this by demonstrating that sharkskin denticle geometries also enhance suction on the suction side of airfoils by promoting small-scale vortex formation. This energizes the boundary layer, prevents premature separation, and improves aerodynamic performance, particularly at low-to-moderate angles of attack. In continuation to this, Bhatia et al. [121] reported that incorporation of sharkskin denticle patterns on NACA0012 airfoil yielded about 4.3% drag reduction and 3.6% increase in lift-to-drag ratio. These effects manifest primarily in the turbulent regime. For instance, riblets can disrupt spanwise turbulent eddies but have little influence under laminar flow [122]. At high Reynolds numbers, sharkskin riblets perform optimally in fully turbulent boundary layers, whereas on laminar surfaces, they confer no benefit and are generally avoided [122,123]. Importantly, they do not adversely affect lift when confined to flow-parallel grooves; in fact, strategic placement can modestly improve lift by maintaining attached flow [121]. Overall, the sharkskin model excels in reducing turbulent drag without need for active flow control, making it a practical aerodynamic enhancement for high-Re flows. In addition to the well-documented role in drag reduction, the functional versatility of sharkskin-inspired surfaces extends to biofouling resistance. Chen et al. [120] investigated this aspect by conducting comparative immersion tests of a biomimetic sharkskin surface and a conventional, smooth surface in an open algal pond environment. The biofouling accumulation on smooth and biomimetic surfaces is shown in Figure 9e,f. Over the course of the exposure period, it was reported that the biomimetic sharkskin surface demonstrated a remarkable 90% reduction in biofouling accumulation compared with the smooth surface. This significant difference is attributed to the microstructured riblet pattern, which disrupts the settlement and adhesion mechanisms of algae and other fouling organisms. The surface topography likely interferes with the ability of bio-organisms to anchor effectively while also potentially creating localized shear conditions that discourage colonization. These findings underscore the multifunctional benefits of sharkskin-inspired designs, which not only enhance aerodynamic and hydrodynamic performance but also offer passive non-chemical antifouling capabilities. This can be particularly valuable for marine and biomedical applications where surface cleanliness and efficiency are critical.

### 3.2. Transition Delay

**Figure 10:**
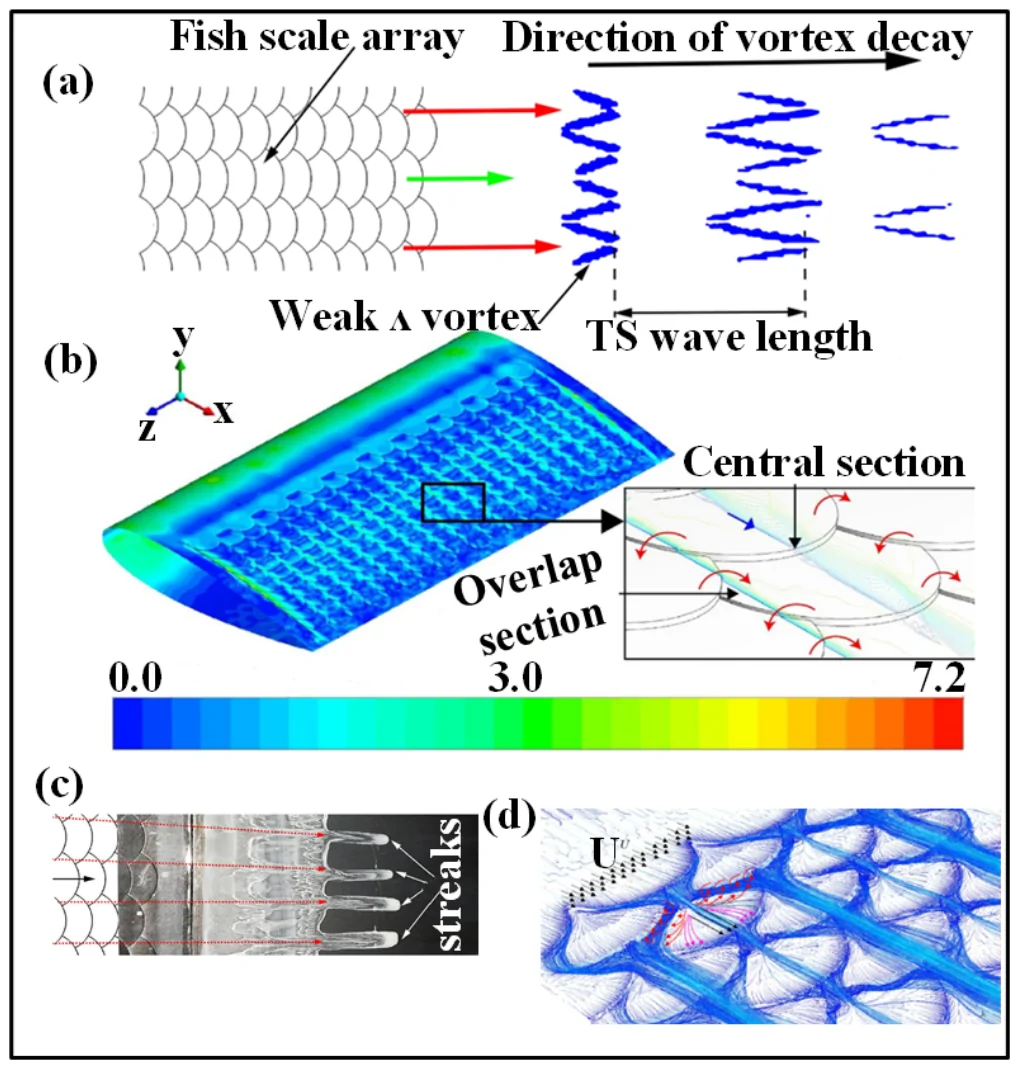
Fluid flow surrounding biomimetic wing inspired by fish scales: (**a**) Flow behind fish scales [42]. (**b**) Flow over fish scale-inspired wing surface [38]. (**c**) Visualization of flow over fish scale surface [37]. (**d**) Flow field in the form of velocity magnitude on the fish scales [39].

In terms of fish scale-inspired surfaces, the overlapping nature of scales introduces localized step changes in boundary-layer thickness and promotes interfacial vortex generation [124]. Natarajan and Rose [38] used 3D-printed fish scale airfoils to show that overlapping ridges interact with incoming flow to induce micro-recirculation zones, as shown in Figure 10. These vortices suppress early transition and facilitate streamlined flow reattachment downstream. Their CFD results confirmed the emergence of alternating high- and low-momentum streaks that support laminarization and minimize overall pressure drag [38]. In continuation to this, Chen et al. [39] suggested that the scale-induced microvortices persist into turbulence, possibly altering the turbulent structures in a favorable way, as depicted in Figure 10d. But the drag reduction in fully turbulent flow is expected to be smaller than in transition delay. Still, even in turbulent boundary layers, the presence of overlapping scale micro-cavities could create drag-reducing low-speed streaks [125]. Recent experiments by Muthukumar et al. [42] using a flat plate covered in artificial fish scales demonstrated a ≥25% reduction in skin friction drag due to delayed transition. The overlapping scales generate a spanwise zig-zag or streak pattern in the flow, which was found to suppress the growth of Tollmien–Schlichting (TS) waves, as presented in Figure 10a [37]. This suppression mechanism is attributed to the modification of the mean velocity profile, where the fish scale pattern induces a spanwise-averaged flow with a steeper velocity gradient near the wall compared with the canonical Blasius boundary layer. The resulting profile exhibits a lower shape factor, a known indicator of increased flow stability and transition resistance.

By passively reshaping the near-wall velocity distribution, the fish scale array effectively prolongs the laminar regime. Conventionally, any surface roughness would accelerate transition. But fish scales, by virtue of their particular size/spacing and the gaps between them, can do the opposite by acting like an array of very small vortex generators [37,42]. This can stabilize the laminar flow. Furthermore, oil-flow visualization on fish scale surface confirmed the presence of orderly streaks aligned with scale geometry, as depicted in Figure 10c, supporting this drag reduction mechanism in nature [37,42,126]. The effect is most pronounced in the laminar- and transitional-flow regimes. Essentially, a fish scale surface maintains laminar flow longer along a surface than a smooth plate would, thus reducing friction over that region [127]. Once the flow is fully turbulent, the benefits of the scale texture may lessen [128]. Notably, Chen et al. [129] carried out a numerical study on a hydrofoil with fish scale texturing, and reported about 10% drag reduction at Re ∼ $4\times 10^{4}$. They further showed that the benefit diminishes if the scale spacing is not optimal. This underscores that scale geometry (size, protrusion angle, and overlap ratio) must be tuned to the flow for maximum effect. Too large scales behave like conventional roughness, and too small scales may have negligible influence.

**Figure 11:**
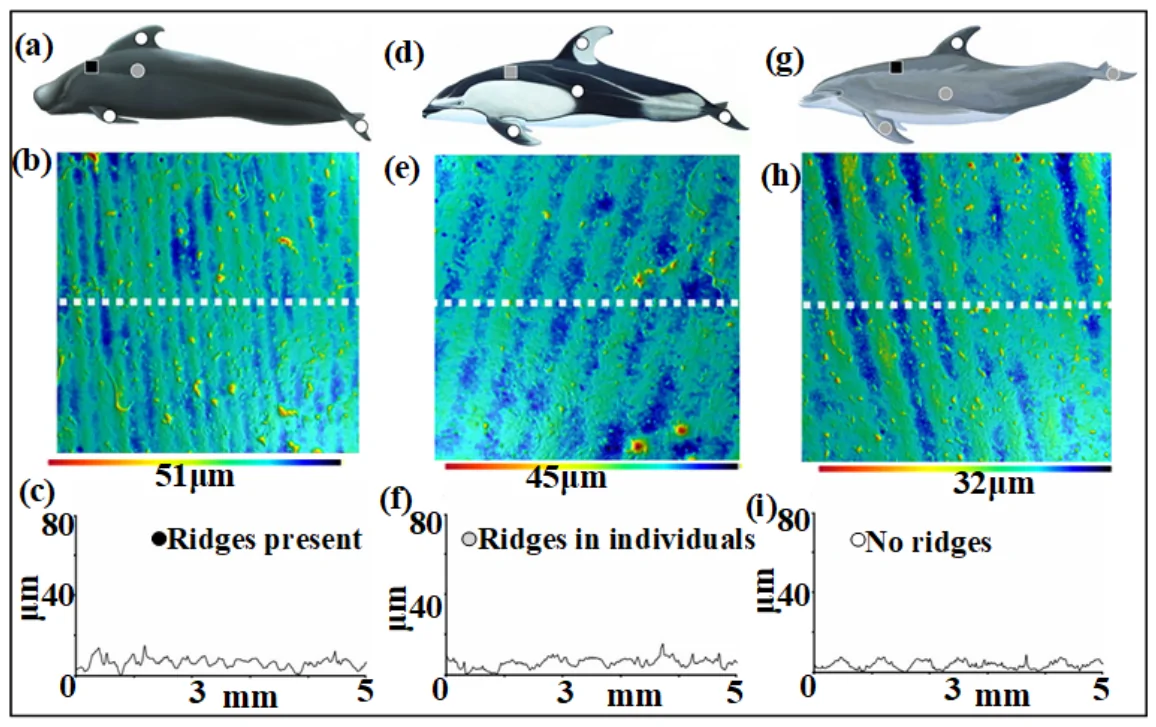
Compliant skin of odontocetes: (**a**–**c**) Pilot whale. (**d**–**f**) White-sided dolphin. (**g**–**i**) Bottlenose dolphin [11].

**Figure 12:**
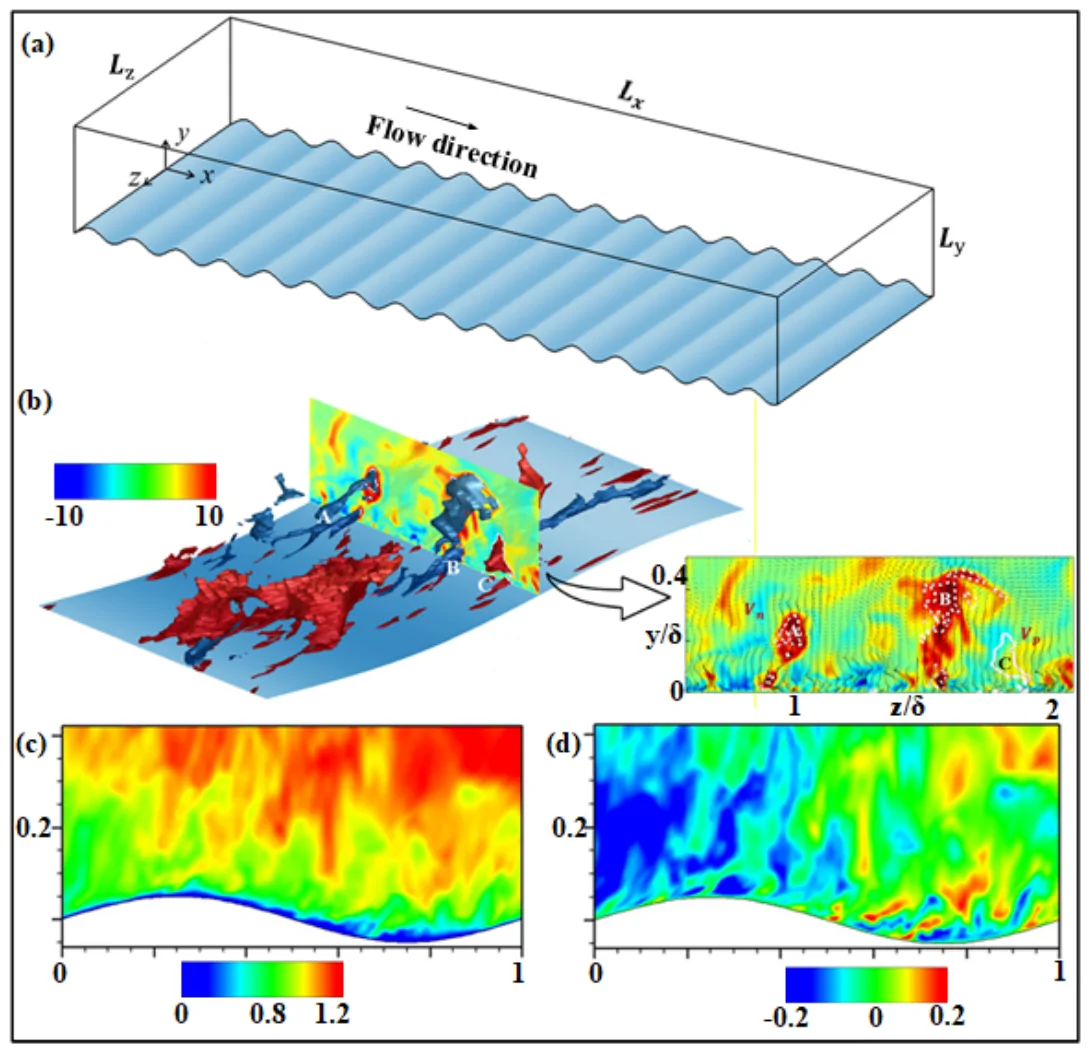
Dolphin-inspired compliant traveling−wave surface: (**a**) Turbulent flow interacting with traveling−wave surface [131]. (**b**) Instantaneous flow field over wavy surface [131]. (**c**) Streamwise velocity [130]. (**d**) Turbulent fluctuation [130] ($L_{x}$, $L_{y}$ and $L_{z}$ are channel length, width and height respectively).

Compliant and dynamic surfaces such as dolphin skin operate via a different mechanism, such as modulation of flow instabilities. The compliant skin of odontocetes is presented in Figure 11 (δ: mesh spacing). Xia et al. [73] examined whether a dolphin-inspired isotropic compliant wall can passively reduce skin friction drag in a fully turbulent boundary layer. Past experiments in transitional flow suggested that compliant coatings might delay transition and reduce drag [74,75,76,79]. But in fully developed turbulence, the DNS revealed that for the tested compliant-wall parameters, the flexible wall actually increased turbulent friction drag rather than reducing it. The wall’s reconfiguration under flow disturbed the boundary layer, enhancing near-wall turbulence and convective momentum transfer, which drove the skin friction coefficient higher compared with a flat wall, as depicted in Figure 12. Thus, instead of stabilizing the flow or damping vortices as intended, the compliant surface intensified turbulence, highlighting the challenges of passive drag reduction via compliant coatings in a turbulent flow. In continuation to this, Xia et al. [81] considers an anisotropic compliant wall to examine whether it can induce beneficial flow patterns to reduce drag. The anisotropic compliant surface did generate a negative Reynolds shear stress very close to the wall as intended, but it did not achieve an overall drag reduction for the cases studied. Instead, the wall’s motion enhanced pressure fluctuation and turbulence in the boundary layer, yielding an overall increase in skin friction drag. The presumed drag reduction mechanism was overwhelmed by increased turbulent momentum exchange, so the compliant anisotropic coating did not lower drag under these conditions. Therefore, compliant surfaces exhibit little to no significant drag reduction effect in fully turbulent flows. Taking the above findings as a reference, Zhang et al. [130] analyzed a moving wavy wall inspired by the compliant skin of dolphins as an active flow-control strategy for drag reduction. They focused on how the traveling-wave motion alters turbulence, especially very-large-scale motions (VLSMs), in the boundary layer. It was reported that the traveling-wavy wall strongly modulates large-scale vortical structures in the flow, with dramatic differences between slow and fast wave motions. At low wave speeds, the wave injects energy into large-scale turbulent motions, lengthening and strengthening the streamwise vortices and roll cells. This increases form drag and turbulence intensity. In contrast, at high wave speeds, the wave motion has an opposite effect. The VLSMs shrink in size and intensity, and the wave–turbulence interaction actually drains energy from the largest scales. Consequently, the mean velocity profile recovers at high wave speeds as the wave-induced form drag diminishes. These results indicate that a fast streamwise traveling wave can stabilize the turbulence and thereby contribute to drag reduction, while a slow or upstream-moving wave destabilizes the flow and increases drag.

**Figure 13:**
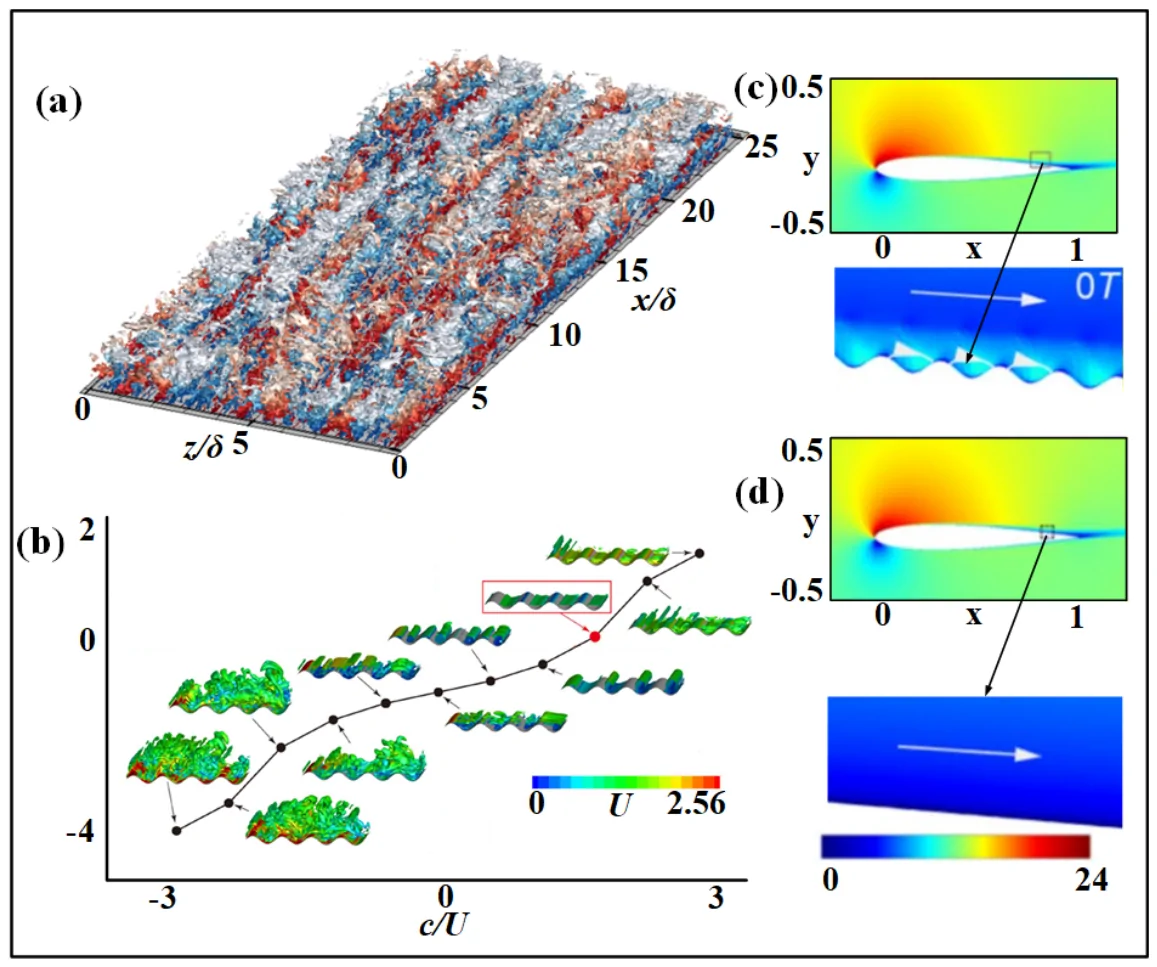
Flow surrounding dolphin skin−inspired compliant surface: (**a**) Iso−surfaces of Q−criterion [133]. (**b**) Flow at different velocity ratios [80]. (**c**) Wavy surface of NACA 0012 airfoil. (**d**) Plain NACA 0012 airfoil [70].

Furthermore, Wang et al. [131] investigated how a traveling-wavy wall alters the hierarchy of wall-attached eddies in turbulence. It was shown that imposing a traveling wave on the wall changes the geometry and energy of turbulent eddies in a scale-dependent way, as shown in Figure 13 (*c* refers to the velocity of the traveling wave). The wavy boundary shortens the streamwise length of attached eddies while widening their spanwise spacing, effectively reconfiguring the turbulence structure. Importantly, for drag, the wave suppresses the small-scale near-wall motions. Additionally, the intensity of near-wall velocity fluctuations drops in the wavy-wall case, indicating weakened streaky structures and sweep events that normally contribute to high friction drag. Meanwhile, larger-scale structures farther from the wall are somewhat enhanced in energy, but they occur in the outer region, where they are less efficient in producing drag. The net effect of a sufficiently fast traveling wave is a boundary layer with damped near-wall turbulence, which can significantly reduce overall friction. Later on, Wang et al. [132] extended the above analysis to scalar transport to examine whether the drag-reducing traveling-wavy-wall configuration can also affect the organization of turbulent scalar fluctuations. It was found that the presence of a traveling-wavy boundary, set to conditions known to alter turbulence, does not destroy the fundamental near-wall transport mechanisms. Meanwhile, in wavy-wall flow, there remains a pronounced near-wall peak in scalar variance and a logarithmic decay in the outer region. This indicates efficient turbulent ejection. Thus, the traveling-wave flow reconfiguration, while aimed at reducing momentum drag, still supports robust scalar mixing through familiar vortex dynamics. In essence, wave-induced drag reduction strategies can be achieved without fundamentally impairing turbulence-driven scalar transport, as the coherent eddy transport mechanisms remain intact in the modified flow.

The latter study conducted by Wang and Liu [70] investigates more about drag reduction using dolphin skin-inspired traveling waves by considering the previous studies as reference. It was reported that the oscillations of dolphin skin were in the form of Longitudinal Micro-Ultrasonic Waves (LMUWs), as shown in Figure 13b [70]. Wang and Liu [70] reported that this may lead to the emergence of a dynamically altered boundary layer. This newly induced near-wall flow structure, as presented in Figure 13c,d, may be associated with a modified force distribution. This can offer substantial potential for pressure drag mitigation and, consequently, overall drag reduction encompassing both pressure and frictional components. As depicted in Figure 13, significant vortex dynamics are predominantly observed during LMUW excitations, especially at elevated oscillation frequencies corresponding to higher absolute values of relative velocity ξ (where ξ=c−U). Here *c* is the speed of the wave, and *U* is the free-stream velocity. When ξ approaches zero, the near-wall vortical structures, including multiple eddies, diminish in strength and eventually dissipate. This may lead to a partially relaminarized and stable flow regime [70]. The most orderly and stable flow is recorded under a downstream-propagating LMUW excitation at 100 kHz, wherein the vortical structures are nearly absent and ξ is minimized. However, with the increase in oscillation frequencies (for instance 200 kHz and 250 kHz) under downstream LMUW, vortical structures re-emerge near the dynamic surface, suggesting re-intensification of the interaction between wall-induced motion and the external flow. These observations emphasize that the parameter ξ critically governs LMUW-induced near-wall flow behavior. When ξ is approximately zero, the LMUW propagation speed closely matches *U*, yielding maximal flow stability. In contrast, a higher relative velocity can accelerate the mismatch between wall motion and ambient flow, thereby enhancing turbulence generation. Unlike Wang and Liu [70], experimental evidence provided by Choi et al. [74] showed that turbulent skin friction decreases in the order of 5–7% with certain silicone-based compliant layers. In continuation to the previous study, experimental replication by Murayama et al. [134] using viscoelastic skin showed a measurable reduction in lift oscillations and delayed stall under dynamic inflow conditions. This behavior stems from compliance-induced damping and phase lag between flow perturbations and skin response. In engineering systems, embedding such surfaces with responsive materials like dielectric elastomers enables similar functionality with active control.

**Figure 14:**
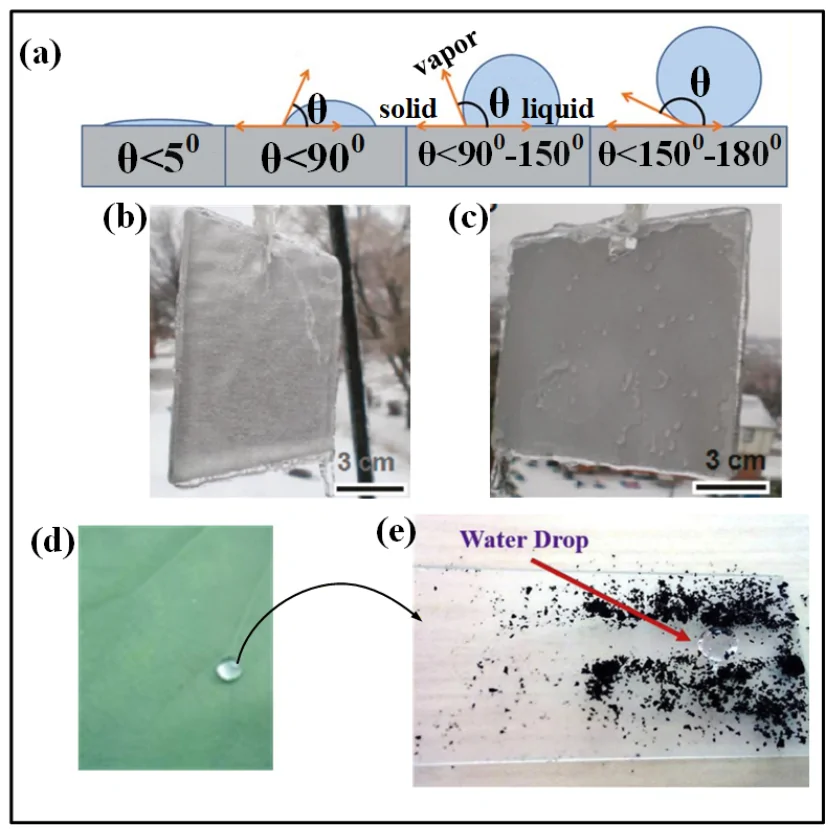
Superhydrophobic surfaces: (**a**) Demonstration of different surface coatings [140]. (**b**) Icing on smooth surface [141]. (**c**) Anti−icing of superhydrophobic surface [141]. (**d**) Water droplet on lotus leaf. (**e**) Demonstration of self−cleaning of coated superhydrophobic surface [55].

A schematic description of hydrophilic to hydrophobic surfaces is given in Figure 14. In the case of superhydrophobic surfaces inspired by lotus leaves, the drag reduction mechanism is primarily the formation of a slip boundary layer. Air pockets entrapped within surface grooves create a Cassie–Baxter state that allows for partial fluid slippage over the surface, as depicted in Figure 4b (θ refers to the contact angle). This reduces viscous drag and minimizes wall shear stress. The numerical work conducted by Guo et al. [53] showed that such surfaces can delay transition and reduce skin friction by up to 20%, particularly under low-Re conditions. However, these effects are sensitive to pressure gradients and breakdown under full submersion or high turbulence, highlighting the need for improved robustness in design. The findings reported in laminar and weakly turbulent water flows show superhydrophobic surfaces reducing drag by 10–30% [135,136,137]. The principle is that micro-textures in the Cassie state (air-filled grooves) minimize the contact area between fluid and solid, effectively lubricating the flow, as presented in Figure 14. However, sustaining this drag reduction under real-world conditions is challenging. In fully developed turbulent flow or at higher pressures, the air film can be stripped away or broken into bubbles, causing the drag reduction to diminish or even turn into drag increase [136]. Indeed, many attempts to achieve enduring drag reduction with superhydrophobic coatings in practical pipes or channels failed once the flow reached a critical speed or the surface became wetted [135,136,137]. Thus, while lotus-inspired surfaces offer remarkable drag reduction potential in theory, their aerodynamic benefits are mainly realized in low-Re or multiphase scenarios or in delaying phenomena like icing, as depicted in Figure 14b,c, rather than in steady high-Re airflows. Latthe et al. [55] demonstrated the self-cleaning ability of superhydrophobic surfaces. They reported that the adhesion between contaminants and the surface is significantly weaker than the interaction between water droplets and the contaminants. As a result, a single water droplet can effectively remove black carbon particles along its path, confirming the coating’s self-cleaning efficiency, as given in Figure 14e. Furthermore, they qualitatively assessed the mechanical durability of the coating through a water jet impact test and an adhesive tape peel test. It was reported that the high-velocity water stream was immediately deflected from the surface, and even sustained localized impacts did not impair the coating or alter its wetting characteristics. This self-cleaning action prevents surface roughening by contaminants and thus helps maintain smooth laminar flow. In aircraft wing, for instance, even thin insect residue or ice can trip the boundary layer from laminar to turbulent, sharply increasing drag, reducing lift and degrading fuel efficiency [138]. Theoretically, a self-cleaning superhydrophobic surface can help to preserve laminar flow and lift-to-drag ratio by shedding such debris. For underwater applications, the trapped air layer on a self-cleaning superhydrophobic hull dramatically reduces liquid–solid contact and skin friction [139]. Therefore, ships and submarines can experience measurable drag reduction and also suffer slower fouling [139]. This self-cleaning superhydrophobic effect can translate into higher aerodynamic/hydrodynamic efficiency with extended maintenance intervals. However, from a practical application point of view, these self-cleaning superhydrophobic coatings can face significant challenges. One of them could be their delicate microstructures, which can be easily damaged by abrasion, pressure or chemical exposure. This may cause the trapped air pockets to collapse and the surface to revert to wetting. Moreover, sustaining a stable air plastron under shear and hydrostatic pressure is difficult. Additionally, the prolonged exposure to UV, salt, and biofilms can chemically degrade the surface chemistry.

### 3.3. Morphological Reconfiguration

**Figure 15:**
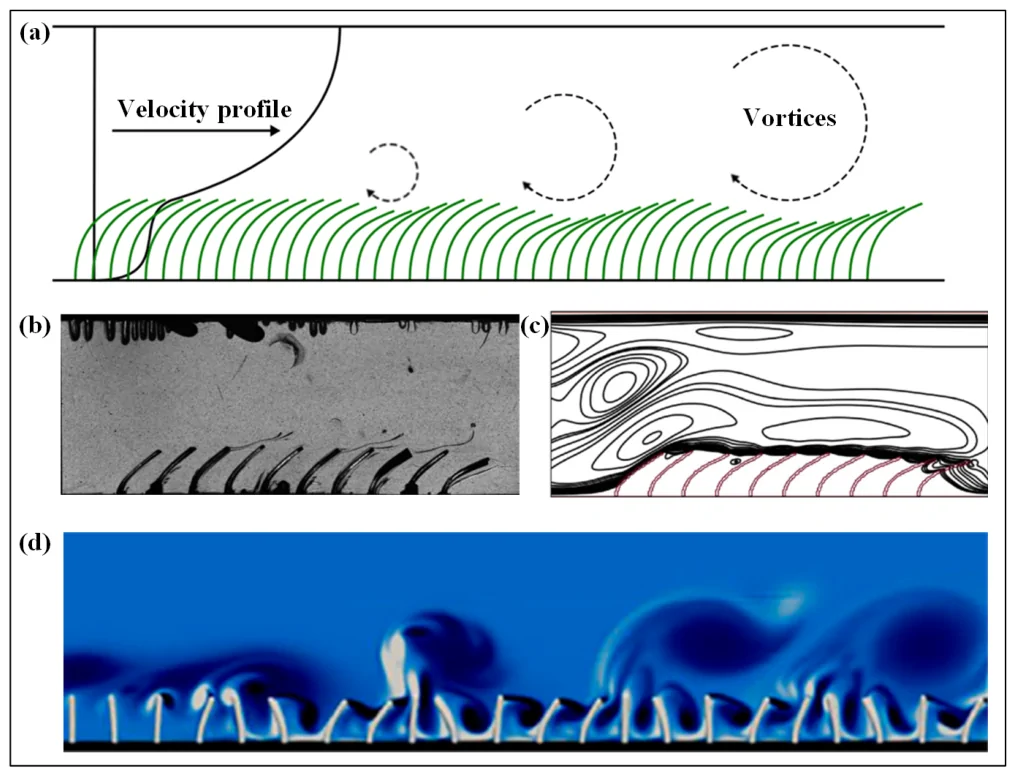
Flow field over flexible filaments mounted on the bottom wall of channel flow: (**a**) Velocity profile of vegetation canopy [144]. (**b**) Experimental velocity field of flexible filaments [89]. (**c**) Instantaneous velocity field of flexible filaments [89]. (**d**) Vorticity contours of flexible filaments [82].

Reconfiguration of flexible filaments and vegetation canopy under flow is one of the most fundamental passive mechanisms for drag reduction. When a slender body such as a filament is mounted in a flow and is sufficiently compliant, hydrodynamic forces bend it downstream. As the flow speed increases, the structure deforms into a more streamlined shape, reducing its effective frontal area and altering wake separation. This passive adjustment to the flow, sometimes referred to as flow-induced shape adaptation, can lower form drag without any external actuation [142]. The physical origin of reconfiguration lies in the balance between fluid loading and structural restoring forces. At low speeds, drag on a near-upright filament scales approximately with the square of velocity ($F_{d}\propto U_{\infty }^{2}$), as in rigid bluff bodies. However, once the bending stiffness is overcome, the body begins to curve. For instance, Alben et al. [142] found that reconfigured filaments follow a Vogel law where drag force is directly proportional to $U^{4/3}$. Subsequent experiments in soap films and numerical simulations confirmed this non-quadratic scaling, illustrating that sufficiently flexible bodies can greatly reduce their drag growth rate with the increase in speed. Beyond this idealized setting, the basic principle holds for a wide variety of flexible geometries. In channel flow, wall-mounted filament arrays reconfigure under Poiseuille or turbulent boundary-layer profiles. For instance, Mao et al. [90] showed that small hairy filaments attached to a cylinder conform into a gently bowed shape under steady flow, reducing the wake recirculation region and cutting form drag by up to 22%. Importantly, their IBM simulations revealed that once a quasi-steady deformed shape is reached, further increase in flow velocity produce only modest additional bending. This saturation effect means that the drag coefficient continues to fall, rather than climbing steeply as for a rigid body. In canopy flows such as laboratory vegetation models, the collective reconfiguration of many elements interacts nonlinearly with the mean shear and turbulence. Monti et al. [85] performed DNS of a turbulent open-channel flow over hundreds of flexible stems and demonstrated that although individual stems bend under local fluid forces, the canopy as a whole retains a coherent wave-like motion, as shown in Figure 15, which was named honami. Crucially, the average canopy height above the wall decreases with the increase in the Reynolds number, effectively shifting the logarithmic velocity profile away from the wall and reducing integrated wall shear stress. This effective slip phenomenon emerges from the reconfigured shape of the canopy, which presents a smoother boundary to the overlying flow and attenuates near-wall turbulence production. Reconfiguration is not just limited to passive steady bending but also encompasses quasi-steady shapes in unsteady or oscillatory flows. In the PELskin oscillatory channel experiments conducted by Favier et al. [89] and Revell et al. [143], silicon flaps attached to the wall exhibited a mean deflection profile superimposed by periodic waving. Even before any dynamic flapping occurs, the flaps first deform into a bent equilibrium under the peak flow velocity. This static component reduces the initial recirculation zone size, lowering form drag at all phases of the oscillation cycle. Only afterward do dynamic oscillations contribute additional drag modification via unsteady wakes and constructive thrust generation. Thus, reconfiguration serves as the first and most robust drag-reducing mechanism, independent of dynamic flutter.

The Cauchy number (Ca), which quantifies the ratio of fluid loading to elastic stiffness, plays a vital role in filament reconfiguration. When Ca<<1, bending is negligible, and the filament behaves rigidly. Similarly, when Ca>>1, the filament is completely aligned with the flow like a flag, offering minimal resistance. In between, at moderate Ca, partial bending yields optimal drag reduction. This deflection is enough to reduce the frontal area. However, sufficient stiffness is required to avoid the filament fully streamlining into a thin, low-leverage shape, which might increase local shear stresses [68]. Experimental studies have mapped out this intermediate regime of maximal reconfiguration benefit, showing that there is an optimal stiffness for a given flow speed and filament geometry at which the drag coefficient is minimized [68,96]. Reconfiguration also depends on aspect ratio and boundary conditions. Doubly clamped filaments with both ends fixed reconfigure differently from cantilevers. The former yield symmetric bending between supports, while the latter exhibit a linearly increasing deflection profile from base to tip [82]. Zhang et al. [87] found that for a doubly clamped filament of fixed span, the maximal curvature and drag reduction occurred at a length equal to roughly two times the spanwise width. In dense filament arrays, element–element interactions alter individual reconfiguration; e.g., neighboring filaments shelter each other. This reduces local fluid loading, thus decreasing individual bending. This collective reconfiguration effect has been observed in PIV experiments on patchy canopies, where inner stems remain straighter than outer ones, producing a gradient in effective boundary height [85]. Material damping and viscoelasticity can modulate reconfiguration. In purely elastic filaments, the equilibrium bent shape under steady flow is static. When filaments exhibit a viscoelastic response, such as time-dependent stress relaxation, the bent shape may evolve slowly over time, leading to an additional drift in drag reduction characteristics. While many laboratory studies use silicone or neoprene with relatively low damping, natural vegetation and engineered polymers often display significant hysteresis and rate-dependent stiffness. Preliminary experiments by Wu et al. [103] on viscoelastic stems showed that under sustained flow, stems creep into a more deflected shape, representing a slow increase in reconfiguration that can further reduce drag but may also lead to structural fatigue. A critical implication of reconfiguration is its robustness across flow regimes. Whether the flow is laminar, transitional, or turbulent, steady reconfiguration occurs whenever fluid forces exceed bending stiffness. Even in high-Re turbulent channel flow, as shown by Monti et al. [85] and Rota et al. [98], the mean stem deflection profile matches that predicted by steady-bending models driven by time-averaged velocity. Instantaneous turbulence-induced fluctuations add dynamic buffeting but do not substantially alter the mean shape responsible for drag reduction. Consequently, passive reconfiguration is a reliable strategy for drag reduction across a wide range of engineering and environmental flows, from microfluidic mixers to riverine vegetation beds. In applied contexts, reconfiguration guides the design of drag-reducing surfaces and bio-inspired devices [145]. For underwater vehicles or pipelines, hair-like coatings of flexible filaments can reduce skin friction at moderate speeds [142,146].

### 3.4. Wake Stabilization

In addition to passive reconfiguration, wake stabilization is a vital mechanism by which flexible filaments reduce drag in channel or bluff-body flows. Unlike reconfiguration, which modifies the body’s instantaneous shape, wake stabilization operates by altering the downstream separation and vortex dynamics, thereby reducing pressure drag and friction losses. This mechanism is particularly effective when flexible elements induce coherent suppression of vortex shedding through passive motion. Wake stabilization emerges when filaments reshape the near-wake region behind bluff bodies or in shear layers, dampening unsteady large-scale vortical structures that typically contribute substantially to drag.

**Figure 16:**
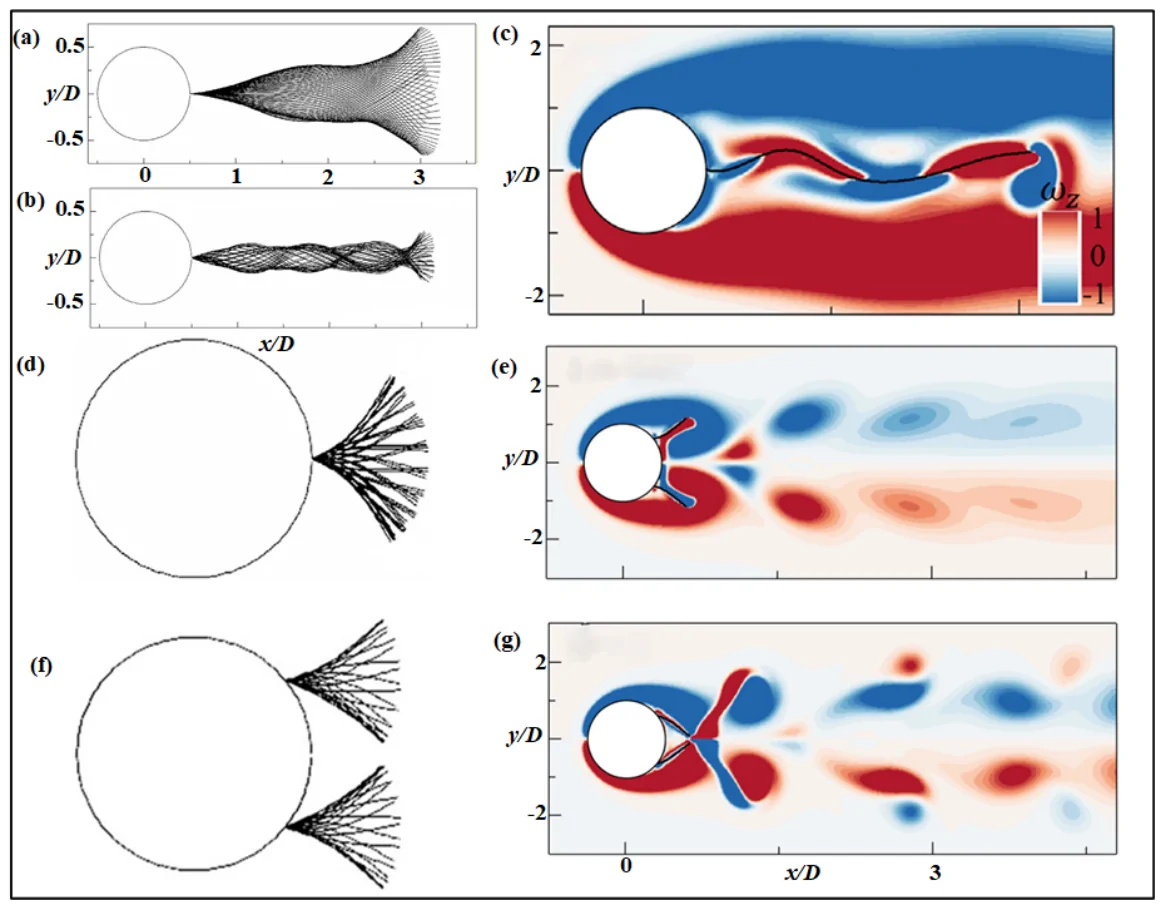
Stationary cylinder with a flapping filament: (**a**) Superimposed filament positions at l=1.0 and pitching angle (α=5°) [90]. (**b**) Superimposed filament positions at l=1.0 and pitching angle (α=25°) [90]. (**c**) Instantaneous vorticity contours at l=1.0 and pitching angle (α=25°) [90]. (**d**) Superimposed filament positions at l=0.5 and α=25° [90]. (**e**) Instantaneous vorticity contour of filaments at extreme outward positions [147]. (**f**) Superimposed 2−filament positions at l=0.5 and α=25° [90]. (**g**) Instantaneous vorticity contour of filaments at extreme inward positions [147].

One of the first demonstrations in this context is the work by Mao et al. [90,147], who attached flexible filaments to the aft portion of a circular cylinder in a laminar channel (Re ∼ 150–200), as depicted in Figure 16 (*D* refers to the cylinder diameter). When filaments were stiff and oscillated according to in-phase snaking motion, they introduced perturbations into the wake but still allowed a classic Karman vortex street to develop. In contrast, out-of-phase clapping motion between two symmetrically mounted filaments disrupted alternating vortex shedding. The left–right symmetry and timing caused destructive interference in successive vortices. This yielded a symmetric wake and reduced form drag by up to ∼15%. Furthermore, it was noticed that the alignment of these filaments increased the effective separation angle, smoothed shear layers, and delayed vortex roll-up. This caused the wake bubble to shrink and numerically reduced pressure drag by up to ∼22%. In this static reconfiguration regime, passive deformation acts as an asymmetric splitter plate, stabilizing shear-layer interaction. If flow speed increases greatly and filaments start to flap vigorously, the effect reverses, where strong oscillatory motion re-energizes wake vortices and actually increases drag [146]. Numerical simulations supported these findings, showing that the wake transforms from a classic 2S Karman pattern into a P+S mode (pair + single vortices), with smaller-amplitude vortices and diminished low-pressure wake [145].

The concept of wake stabilization is still valid for wall-mounted filaments, which modify flow separation and turbulence near the wall. In turbulent open-channel flow over a dense flexible canopy, Monti et al. [85] demonstrated through DNS that compliant stems suppress vertical turbulent fluxes and break up roller-like structures near the canopy edge. These stems bend and sway in response to large-scale streaks but do not generate large coherent wakes themselves. Instead, their motion interrupts the formation of stronger turbulent ejections from the wall, thereby attenuating Reynolds shear stress near the wall. As a result, wall shear decreases, boundary-layer turbulent production weakens, and total drag is reduced. Similar effects were also noticed in oscillatory boundary layer flows over wall-mounted filaments in the PELskin experiments [89]. The silicon flaps on the channel wall deform under oscillating flow, initially into a bent average profile, and then wave synchronously with the flow [143]. It was also reported that the mid-phase deformation systematically suppresses the formation of large recirculation zones adjacent to the wall and smoothens the flow reattachment process. This further reduces the pressure fluctuations near walls, which in turn suppress high-frequency friction drag components. The PIV measurements confirmed smoother velocity profiles above the flap array and reduced pressure drag, which was induced by recirculation, compared with equivalent rigid flap beds. Even in cases of small-amplitude oscillation, the flap motion, which is out of phase with the fluid shear, efficiently suppresses oscillating flow separation.

Another illustration of wake stabilization appears in vegetation canopy experiments under steady flow. Wu et al. [103] conducted flume experiments on flexible aquatic plant beds representing submerged stems. They found that as flow velocity increased, stems bent and partially aligned with the flow but also oscillated gently, intermittently shedding coherent vortices that re-energized flow within the canopy. This helped to maintain a uniform shear layer at the canopy top and prevented formation and dissipation of large vortices. This resulted in reduced pressure fluctuation and lower dynamic drag on the canopy floor. The PIV measurements also demonstrated that flexibility stabilizes the shearing interface compared with a rigid array of filaments. A broader perspective is provided by horizontal to vertical filament arrays in channel flows. Arrays of short articulated flaps were attached along the channel floor. It was reported that this arrangement acted as micro-geometry shock absorbers that broke up incoming turbulence before it impinged downstream. In experiments by Singh et al. [68] in a large channel with wall-mounted flexible posts, PIV and tracer dispersion methods revealed that flow behind the posts reattaches rapidly and avoids forming extended separation zones. Even at moderate Re (Re ∼ 2000), coherent wake behind individual posts were attenuated by downstream filaments, leading to smoother mean flow and reduced drag accumulation. As the filaments moved passively in response to unsteady shear, the entire bed behaved like a porous foamy buffer zone. It was reported that this zone stabilized wake formation and reduced pressure drag, while simultaneously enhancing mixing. It is instructive to compare these passive stabilization mechanisms with active control.

Rigid splitter plates or trailing edges can stabilize flow and reduce drag, but they impose a fixed cross-section and cannot adapt to changing flow speeds. Flexible filaments, in contrast, tailor the shear-layer interface dynamically. At low speeds they retain moderate stiffness and provide some bluffness. At moderate speeds they bend and subtly alter the shear-zone geometry. At higher speeds they yield further and narrow the wake with reduced recirculation. This self-adapting stabilization thus offers drag reduction over a wide operating range. Quantifying the wake-stabilization effect in simulations demands fine resolution of the wake vortex field and fluid–structure interaction. DNS studies by Rota et al. [98] examined turbulent channel flow over flexible cane-like stems and compared two cases, namely, stems allowed to sway and stems frozen in an instantaneous bent shape. The frozen stems with no motion yielded lower total drag than the swinging stems. This indicates that steady reconfiguration is more effective than unsteady flapping in wake suppression. TKE around oscillating stems was higher in the fully flexible case, leading to additional turbulent drag. Importantly, the reduction in wall shear and pressure drag in the frozen case arises precisely due to wake stabilization through steady bending. In field-scale analogs, vegetation swaying in riverine flows demonstrates wake stabilization on larger scales. Studies of submerged meadows of aquatic plants show that under normal current, stems oscillate in resonance with ambient wakes, but when storms or pulses increase, stems bend and remain in a steady streamlined posture., thus allowing for faster reattachment, reduced wake length, and mitigated drag on channel beds. While these natural flows are complex and three-dimensional, the underlying stabilization mechanism that reduces wake recirculation is consistent with laboratory and numerical observations.

When the flapping frequency of flexible filaments diverges from that of natural vortex shedding, phase decoupling occurs. In clapping filaments, this leads to asymmetric vortex morphologies that cancel classical alternating shedding. Similarly, in hairy coatings, the bowed gap between stiff base and tip filament acts like a flow splitter that forces separation to occur further downstream or more gradually, reducing wake pressure deficits. In turbulent canopies, the stabilized wake zone near the canopy top produces a smoother velocity gradient above and a thinner region of shear production below, suppressing sweep–ejection cycles and altering friction drag partition. One subtle yet important consideration is the role of mass ratio and inertia. A very light filament may flutter at high frequency, energizing wake and yielding little net stabilization. A very heavy filament, on the other hand, may be too rigid or inertial to respond adequately. Thus, optimal wake stabilization often requires a moderate mass ratio light enough to bend and align, yet stiff and inertial enough to resist rapid flapping. Mao et al. [90] observed that drag-reducing clapping motion only occurred within a narrow mass ratio window. Outside this window, filaments either flapped uncontrollably or remained too straight to affect vortex formation. Viscous damping and structural damping also influence wake stabilization. In experiments by Wu et al. [103], stems with higher internal damping deformed slowly and stabilized wakes more consistently than lightly damped stems. While extensive flapping can temporarily break wake structures, damping can dissipate unwanted oscillations and allows for steady reconfigured shapes to guide wake recovery. Similarly, canopy stems with viscoelastic materials exhibited sustained steady alignment during long-duration flows, reducing fluctuation-based drag.

### 3.5. Flow Deceleration

Another important drag reduction mechanism provided by compliant filament structures in channel flows is flow deceleration. It is the ability of flexible filaments to slow down adjacent fluid layers and thereby reduce turbulent convective momentum transfer and skin friction. While reconfiguration focuses on shape adaptation and wake stabilization on vortex suppression, flow deceleration emphasizes the modification of the local velocity field.

**Figure 17:**
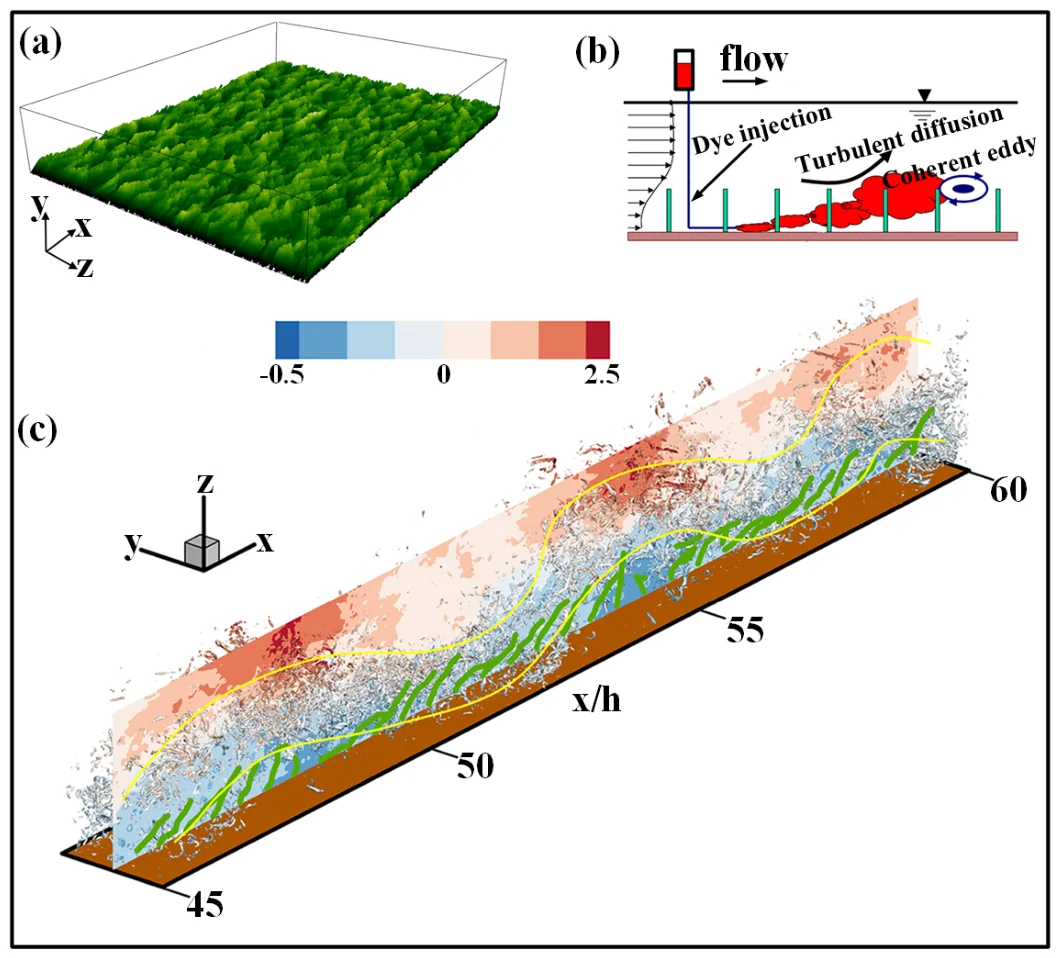
Flexible filaments: (**a**) Canopy exhibiting wavy motion [98]. (**b**) Streamwise velocity fluctuations over filaments [148]. (**c**) Q-criterion combined with streamwise velocity fluctuations [96].

As filaments bend or sway, they alter the velocity gradient near walls, creating buffer regions that reduce mean shear stress, especially within turbulent boundary layers, as depicted in Figure 17. Physically, when filaments bend and partially align with the flow, they disrupt the high velocity gradients typically found close to solid surfaces. In a turbulent open-channel flow over a flexible canopy, high-resolution DNS with flexible stems shows that as flexibility increases, the stems penetrate further into the mean flow and reduce the shear-layer thickness at the canopy top [85]. This deceleration of near-bed streamwise velocities can effectively shift the log-law region away from the wall. Since shear stress at the wall depends on the gradient of mean velocity at the surface, a reduced gradient translates directly into lower wall shear stress. This can be expected to decrease drag. Rota et al. [98] also reported that freezing the canopy in a static bent configuration can further reduce integrated wall shear. This confirms that a steadier flow deceleration region is more effective in drag reduction than unsteady flapping that re-energizes turbulence via wake pumping [98]. Experimental findings provided by Wang et al. [96] complement these findings. The PIV measurements show that bent flexible stems produce a smoother vertical velocity profile through the canopy. The peak velocities occur higher above the bed, while interior canopy flow is subdued. This decelerated region acts like a buffer, reducing the extent and intensity of ejection events that would otherwise transport momentum downwards and raise drag. As stems reconfigure with the increase in flow speed, the buffer can grow thicker. This may reduce Reynolds shear production and thus skin friction and pressure drag in combination. At the filament-array level, Singh et al. [68] found that arrays of moderately flexible filaments spaced evenly across the channel floor yielded a decelerated near-wall region. Instead of sharp shear layers near the wall, flow decelerated gradually through the hairy layer and only reached free-stream velocity above the filament tips. This slower region reduces the convective momentum transfer between high-speed flow and the bed, effectively damping turbulent sweeps and ejections induced by strong wall-normal velocity fluctuations. As a result, time-averaged wall shear stress was reduced by several percentage points compared with both flat-wall and rigid-filament cases.

The DNS studies by Rota et al. [98] showed that for stems of moderate flexibility, turbulent shear decreases significantly near the canopy tip and inside the canopy but viscous shear remains relatively small. Consequently, the major drag reduction stems from reduced Reynolds shear, itself a consequence of flow deceleration in the stem-influenced region. A broad region of spatially constant velocity can be created when stems are frozen in a steady bent shape. This effect can be even stronger than flapping flexible stems, because turbulence production from stem motion is suppressed. Flow deceleration is also a self-regulating phenomenon. As flow speed increases, filaments bend further, deepening the deceleration region, which in turn reduces local flow speeds and dampens turbulent energy production. Thus, the system tends toward a quasi equilibrium where fluid forces and filament shape achieve a balance. This regulatory outcome depends critically on filament material and stiffness. Highly stiff filaments fail to decelerate the flow effectively, whereas highly flexible or lightweight ones may bend almost flush with the wall, creating a nearly impermeable layer that blocks flow but also prevents beneficial mixing. Optimal drag reduction typically occurs at moderate flexibility and density. Experimental observations confirmed that infinite flexibility can suppress velocity gradients too strongly, leading to increased upstream pressure or static friction and thus diminishing net drag benefits [89]. Flow deceleration mechanisms also interact with canopy-scale coherent motions. Honami and monami waves can generate time-varying flow fields that can intermittently energize velocity fluctuations. However, when reconfiguration dominates and steady bending prevails, the deceleration region remains thick and stable. For instance, in comparative studies of oscillatory and steady stems, the steady deflection component contributes the most to drag reduction, whereas the dynamic wave component contributes more to mixing but less to sustained deceleration. Experiments in oscillatory flap arrays revealed that monami increases mixing and the average reduction in shear drag comes primarily from upstream bending, which creates an extended low-velocity region. Flow deceleration is further supported by visual measurements of velocity fields. In both canopy and filament-array studies, instantaneous PIV snapshots show high-velocity core flow well above the elements, separated from the bed-proximate wake and walls by a slower, shear-limited layer. The gradient across that intermediate region is much less steep than in smooth-wall channel flows. The time-averaged velocity profiles revealed that the mean velocities near the bed are subdued with a decrease in the shape factor of the boundary layer. These indicators show that flow deceleration is both measurable and relevant to performance improvements in drag reduction.

## 4. Limitations, Research Gaps, and Future Directions

Despite the promising progress in the development and implementation of bio-inspired fine structures, several limitations persist that hinder their full-scale deployment in aerospace, automotive, and marine applications. Understanding these limitations is essential to advancing the field and identifying fertile ground for future research. One of the key challenges lies in scaling and manufacturability. Many bio-inspired surfaces are fabricated at micro- or nano-scales using techniques such as Nano-Imprint Lithography (NIL), soft lithography, and laser ablation [149,150,151]. While these methods enable precise reproduction of natural patterns, they are often costly, time-intensive, and difficult to scale for large, contoured surfaces such as aircraft wings or marine hulls. It should be noted that the drag reduction values reported in the literature are predominantly obtained under idealized laboratory conditions, which may differ substantially from the complex environments encountered in practical applications.

For instance, most sharkskin-inspired designs rely on uniform riblets or denticle patterns, whereas actual sharkskin features variation in scale, orientation, and compliance that contributes significantly to its effectiveness [25]. Similarly, fish scale arrays used in artificial contexts often ignore the dynamic flexibility and mucus-lubricated interface found in real fish [49]. Another limitation is the narrow operating range of many bio-inspired structures. Riblets, for instance, provide drag reduction within specific ranges of Reynolds numbers and angles of attack [33]. Outside of these operating conditions, their effectiveness either diminishes or may even lead to increased drag. Similarly, fish scale-inspired surfaces and feather-based flaps must be carefully tuned in terms of spacing, orientation, and flexibility to maintain performance across varying aerodynamic loads. Implementing dolphin skin concepts also faces considerable practical challenges. Passive compliant coatings can be applied over large surfaces, but crafting a material with the right viscoelastic response across a range of frequencies is difficult to implement. However, the flow regimes targeted by such methods are turbulent, where there is ample chaotic energy to suppress. At low Re or laminar flow, a compliant surface could suppress instabilities and the flow stays attached for a longer duration. In a transitional-flow regime, a properly tuned compliant surface might delay transition by damping disturbance growth, similar to fish scale effects, but this requires precise control. In essence, dolphin skin strategies are most relevant from the upper end of laminar regimes to transitional-flow regimes. But the drag reduction capabilities of compliant surfaces in fully turbulent flow are still unclear.

Furthermore, the drag reduction observed in laminar- or transitional-flow regimes may not hold under fully turbulent or high-speed conditions in typical aerospace and automotive applications [152]. Superhydrophobic surfaces and flexible filaments, for instance, lose effectiveness when submerged for long periods or subjected to shear forces, making them unsuitable for some marine applications [153]. Additionally, passive systems lack adaptability, making them vulnerable to performance degradation when flow conditions deviate from those for which they were optimized [154]. From a fabrication perspective, current methods often fail to capture the multi-scale hierarchy found in natural surfaces. Hierarchical textures combining nano- and micro-scale features are particularly challenging to reproduce in large-scale curved geometries [70]. Moreover, the materials used in synthetic systems do not yet match the multifunctionality and durability of biological counterparts, particularly with regard to self-healing, biofouling resistance, or environmental responsiveness [135,155]. Finally, a critical research gap lies in the integrated study of bio-inspired systems. Many investigations focus on single structures or isolated effects, whereas natural organisms leverage multiple mechanisms for optimal performance. Moving forward, interdisciplinary research is needed to develop multifunctional, hybrid surfaces that incorporate drag reduction, sensing, and structural compliance in a unified design. Additionally, integration of these surfaces with existing structural materials without compromising aerodynamic or structural integrity remains an open problem. Another notable limitation is durability and environmental resilience. Natural surfaces operate in self-regenerating biological systems, which continuously repair or shed damaged parts. In contrast, synthetic biomimetic materials lack self-healing properties and are prone to mechanical wear, fouling, and performance degradation under UV exposure, temperature cycling, or high-speed abrasion.

Modeling and predictive limitations also constrain advancement. Despite rapid growth in CFD, LES, and fluid–structural interaction (FSI) tools, there remains a discrepancy between simulated and experimental outcomes, especially for flexible, dynamic, or morphing surfaces [122,156]. These discrepancies arise from simplifications in material behavior, flow assumptions such as incompressibility or laminar-turbulent transition models, and boundary conditions. As dynamic surfaces become more intelligent and integrated with sensors and actuators, accurate multiphysics modeling becomes increasingly critical. In terms of smart and responsive systems, bio-inspired surfaces that incorporate feedback mechanisms, such as artificial hair sensors or covert-feather-based flaps, introduce new challenges in electronics integration, power management, and data interpretation [157]. These systems often require real-time control loops and robust signal processing, which must be fault-tolerant, lightweight, and energy-efficient. Furthermore, developing algorithms that translate flow information into actionable surface responses, such as morphing or stiffness modulation, is still in early stages [122,158]. Beyond technical challenges, there are also standardization and testing issues. Current methodologies for evaluating drag reduction performance vary widely across studies, with differences in tunnel conditions, flow metrics, and reporting standards. This variability makes it difficult to compare different approaches or benchmark performance across bio-inspired strategies. A unified testing framework and standardized protocols would enhance reproducibility and facilitate systematic comparisons. Looking ahead, future research directions should emphasize multifunctionality and adaptability. Integration of multiple surface functionalities such as combining riblets with flexible filaments or embedding sensors within compliant surfaces can offer promising output for aircraft and marine vehicles that operate in dynamic, uncertain environments. Another promising direction could be developing new materials that can adapt the self-healing properties of natural surfaces. This approach could significantly enhance the robustness, longevity, and environmental compatibility of bio-inspired designs.

## 5. Conclusions

The study of bio-inspired surface and morphological structures for aerodynamic and hydrodynamic performance enhancements represents a remarkable intersection of biology, fluid dynamics and engineering design. This review has comprehensively covered several biological sources of inspiration, namely, sharkskin denticles, fish scales, dolphin skin, superhydrophopic surfaces and vegetation canopies. The body of evidence in experimental and numerical studies shows that biomimetic structures can significantly improve aerodynamic and hydrodynamic performance when carefully designed and tuned to specific operational environments.

Sharkskin-inspired riblets, for instance, have demonstrated significant turbulent momentum exchange, yielding reduction in skin friction drag typically in the order of 10–30% under controlled conditions. Furthermore, these riblets act as micro-flaps that prevent flow reversal and delay boundary-layer separation. In essence, textured shark skin combines frictional drag reduction and vortex suppression to maintain streamlined flow attachment. Fish scales, arranged in imbricated overlapping arrays, offer a different drag reduction strategy. The slight protrusions and spacing between scales generate a streaky boundary-layer flow. This zig-zag micro-flow structure disrupts the growth of Tollmien–Schlichting waves and delays the laminar-to-turbulent transition. Experiments have demonstrated over 27% skin friction drag reduction by distributing fish scale arrays on plain surfaces under laboratory conditions by maintaining a more orderly laminar-flow regime over the surface. Compliant dolphin skin highlights yet another mechanism. The mechanism involves smoothing out small vortex perturbations. As the compliant skin yields to flow, it attenuates instabilities and even forms gentle wave-like bulges. These micro-deformations can entrain tiny vortices that serve as a buffer layer, further diminishing drag. It was reported that surfaces inspired by dolphin skin can postpone transition and reduce near-wall turbulent intensity, yielding substantial skin friction drag reduction of up to 49% in controlled experiments. Nevertheless, the capability of compliant surfaces to reduce drag in fully developed wall turbulence is still a controversial issue according to recent high-fidelity numerical simulations. The drag reduction mechanism of superhydrophobic surfaces is mainly the formation of a slip boundary layer. This mechanism does not necessarily alter turbulence in the bulk flow but minimizes viscous drag at the surface itself, offering a substantial reduction in frictional resistance. Studies inspired by superhydrophobic surfaces reported drag reduction of up to 77% under low-Re conditions. However, the effectiveness of these surfaces can be significantly reduced under high-speed flows. Finally, flexible-filament-based vegetation canopies on plain surfaces have been shown to achieve drag reduction through flow-aligned reconfiguration. This reconfiguration leads to a more uniform, less turbulent flow within and right behind the canopy. The filaments self-adjust into a shape that delays vortex formation and stabilizes the wake region, providing passive control over the flow. This principle has even inspired engineered hairy coatings on cylinders and marine structures. When tuned correctly, such coatings form a stable deformation that calms the recirculating flow and cuts down pressure drag by over 50%.

Overall, these biological systems demonstrate a spectrum of drag reduction mechanisms under the unifying theme of flow control. Each solution targets a different aspect of fluid–structure interaction. The convergence of these strategies provides inspiring design paradigms for engineering applications.

## Figures and Tables

**Table 1:** Summary of sharkskin-inspired drag reduction.

Morphology	Conditions	Mechanism	Drag Reduction
Sawtooth riblets [24]	$Re=3\times 10^{4}$	Protruding height theory	1.53% (LES)
Scalloped riblets [24]	$Re=3\times 10^{4}$	Protruding height theory	12.02% (LES)
Hybrid riblets [24]	$Re=3\times 10^{4}$	Protruding height theory	14.11% (LES)
Denticles [25,26]	$Re=4\times 10^{4}$	Delay of flow separation	3–10% (direct force measurement)
Shortfin mako shark [27]	$Re=28\times 10^{2}$	Control of flow separation	—
Flexible sharkskin foil [28]	$Re=9\times 10^{4}$	Enhanced suction	—
Riblet-structured coating [18]	Oil film channel	Wall shear stress reduction	—
Sharkskin micro-grooves [29]	Water tunnel test	Vortex weakening	—

**Table 6:** Comparison of bio-inspired surfaces for aerodynamic and hydrodynamic performance enhancement.

Bio-Inspired Surface	Biological Inspiration	Primary Mechanisms	Drag Reduction
Sharkskin	Riblets Denticles Grooves	Enhanced suction Secondary vortex Height theory	≤15% [24,26,27,28]
Fish scales	Overlap scales Mucus Fins	T-S wave attenuation VIV mitigation Viscous and elastic theories	≤27% [37,41,42,43,44,110]
Compliant dolphin skin	Viscoelastic skin Oscillating surface	Delay of transition Laminar-flow stabilization Fluid damping suppression	≤49% [74,75,76,79]
Superhydrophobic surfaces	Lotus leaf Rice leaf Non-wetting surfaces	Slip velocity Anisotropic flow Frictional resistance suppression	≤80% [60,61,62]
Flexible filament arrays	Vegetation canopy Hairy surface	Reconfiguration Wake stabilization Flow deceleration	≤50% [109,111]

## Data Availability

The data that support the findings of this study are available from the corresponding author upon reasonable request.
